# Molecular mechanisms of NET formation and degradation revealed by intravital imaging in the liver vasculature

**DOI:** 10.1038/ncomms7673

**Published:** 2015-03-26

**Authors:** Elzbieta Kolaczkowska, Craig N. Jenne, Bas G. J. Surewaard, Ajitha Thanabalasuriar, Woo-Yong Lee, Maria-Jesus Sanz, Kerri Mowen, Ghislain Opdenakker, Paul Kubes

**Affiliations:** 1 Department of Physiology and Pharmacology, Calvin, Phoebe and Joan Snyder Institute for Chronic Diseases, University of Calgary, HRIC 3280 Hospital Drive N.W., Calgary, Alberta, Canada T2N 4N1; 2 Department of Evolutionary Immunology, Institute of Zoology, Jagiellonian University, ul. Gronostajowa 9, 30–387 Krakow, Poland; 3 Laboratory of Immunobiology, Rega Institute for Medical Research, KU Leuven - University of Leuven, Minderbroedersstraat 10 blok x - bus 1030, 3000 Leuven, Belgium; 4 Department of Microbiology, Immunology and Infectious Diseases, Calvin, Phoebe and Joan Snyder Institute for Chronic Diseases, University of Calgary, HRIC 3280 Hospital Drive N.W., Calgary, Alberta, Canada T2N 4N1; 5 Department of Pharmacology, Faculty of Medicine, University of Valencia, Av. Blasco Ibañez 15, 46010 Valencia, Spain; 6 Department of Chemical Physiology, Scripps Research Institute La Jolla, 10550 North Torrey Pines Road La Jolla, 92037 California, USA; 7 Department Immunology and Microbial Sciences, Scripps Research Institute La Jolla, 10550 North Torrey Pines Road La Jolla, 92037 California, USA

## Abstract

Neutrophil extracellular traps (NETs) composed of DNA decorated with histones and proteases trap and kill bacteria but also injure host tissue. Here we show that during a bloodstream infection with methicillin-resistant *Staphylococcus aureus*, the majority of bacteria are sequestered immediately by hepatic Kupffer cells, resulting in transient increases in liver enzymes, focal ischaemic areas and a robust neutrophil infiltration into the liver. The neutrophils release NETs into the liver vasculature, which remain anchored to the vascular wall via von Willebrand factor and reveal significant neutrophil elastase (NE) proteolytic activity. Importantly, DNase although very effective at DNA removal, and somewhat effective at inhibiting NE proteolytic activity, fails to remove the majority of histones from the vessel wall and only partly reduces injury. By contrast, inhibition of NET production as modelled by PAD4-deficiency, or prevention of NET formation and proteolytic activity as modelled in NE^−/−^ mice prevent collateral host tissue damage.

## Abstract (web summary)

Neutrophil extracellular traps (NETs) released by neutrophils trap pathogens but may also cause tissue damage. Here the authors show that during systemic *Staphylococcus aureus* infection NETs anchoring to the vasculature are only partially DNase-sensitive, advocating for better anti-NET therapies.

## Graphical abstract

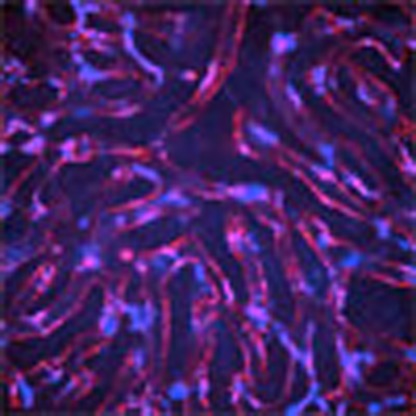

Neutrophils are among the first non-resident, immune cell responders of early infections^1^. These cells rapidly react to infection and clear pathogens by various means including phagocytosis, antimicrobial killing by factors released during degranulation or through the formation of neutrophil extracellular traps (NETs)^2^,^3^. These extracellular DNA structures were discovered just a decade ago and their primary function has been delineated to immobilize and kill pathogens^4^. Within the bloodstream, the capture of the pathogens, for the most part, remains a function of Kupffer cells within the liver vasculature. These cells express a specialized pathogen receptor, the Complement Receptor of the Immunoglobulin superfamily (CRIg), which has evolved to catch circulating pathogens under shear conditions^5^. By contrast, neutrophils are not capable of directly catching circulating pathogens but by producing NETs, they can increase the catching capacity of the liver^6^. In addition, NETs may also have lasting effects by modulating tissue healing and even shaping the late (adaptive) immune response^1^,^7^.

The enzymes, peptidyl arginine deiminase type IV (PAD4) and neutrophil elastase (NE), have been implicated in the initial decondensation of DNA and the proteolytic degradation of the nuclear envelope^8^,^9^. The DNA is then released through lysis^10^, vesicular transport and degranulation^11^ or by some as yet unresolved catapulting mechanism^3^. Regardless, the negatively charged DNA functions as the backbone of the NET and binds the other NET components, potentially through positive electrostatic charge^4^. These additional NET components include nuclear proteins (histones), cytoskeletal proteins and granular proteins, including proteases^4^. DNase has been shown to completely dissolve NETs *in vitro* 4, and is consequently used both in animal models^12^ and perhaps even clinically^13^,^14^ to remove NETs.

Because of the cytotoxic mix of proteins and enzymes bound to NETs, these structures have been described as a double-edged sword, not only facilitating pathogen elimination, but also eliciting damage to bystander cells^6^,^15^. Pathogenicity of NETs has also been implicated in the promotion of thrombosis as NETs serve as scaffolds for fibrin, von Willebrand factor (VWF) and thrombus formation^16^. Although histones have a potent antimicrobial action^17^, they can also damage and kill endothelial and epithelial cells^18^,^19^. Another potentially harmful molecule is the potent broad-spectrum protease NE^4^,^20^. A single drop of plasma has an anti-proteolytic shield that can inactivate the elastase released from millions of neutrophils, however, when elastase was bound to DNA it preserved NE activity, as was shown long before NETs were described^21^,^22^,^23^,^24^. This implies that NETs might be reservoirs of active, inhibitor-insensitive NE, and DNase could be unleashing a proteolytic storm into the bloodstream, or could simply breakdown the NETs. However, even this latter point, that is, are all NET components removed with DNase, is really not known. These questions have significant clinical relevance as DNase is given as a therapeutic agent, for example, in cystic fibrosis patients^25^ and further clinical applications may be forthcoming.

*S. aureus* bacteremia is one of the most common serious bacterial infections worldwide. In the United Kingdom alone around 12,500 cases each year are reported with an associated 30% mortality^26^. Of 6,697 bloodstream infections from 59 hospitals in the United States of America, *S. aureus* was the most common bacterial isolate, accounting for 23% of all episodes^26^. Although skin and soft tissue lesions are quite common and still treatable, in many patients these infections lead to bacteremia and all too often sepsis^27^,^28^. Furthermore, catheter and heart valve colonization by *S. aureus* also leads to bacteremia^29^ and methicillin-resistant S. *aureus* (MRSA) is becoming a very significant problem in this regard. *S. aureus* appears to be one of the most potent inducers of NETs^11^ both in humans and mice but whether this contributes to improved health or disease remains unclear. To study NET production, persistence and biological importance in *S. aureus* bacteremia, we used multi-laser spinning disk confocal microscopy. We detected MRSA-induced NETs and their associated components (histones and NE). We demonstrate that intravenous infection with MRSA, as happens in catheter infections, vegetative growths on heart valves or in serious skin infections, leads to rapid sequestration of the pathogen to the liver and a neutrophil-dependent NET formation within the liver sinusoids. This neutrophil recruitment and subsequent NET release is associated with profound liver injury. The development of a novel *in vivo* zymography assay revealed that NE bound to DNA is protected from neutralization by plasma. Inhibition of NE activity lining endothelium significantly limited collateral damage. Removal of DNA with DNase failed to remove all histones or proteases from the vascular wall, and provided less protection than prevention of NET formation (NE^−/−^ and PAD4^−/−^ mice). This was because NETs anchored to the vascular wall, in part via VWF. Our data indicate that the effectiveness of DNase might be limited in terms of removal of the most dangerous NET components and advocates for inhibition of NET production.

## Results

### MRSA homes primarily to the liver leading to tissue damage

**Figure 1:**
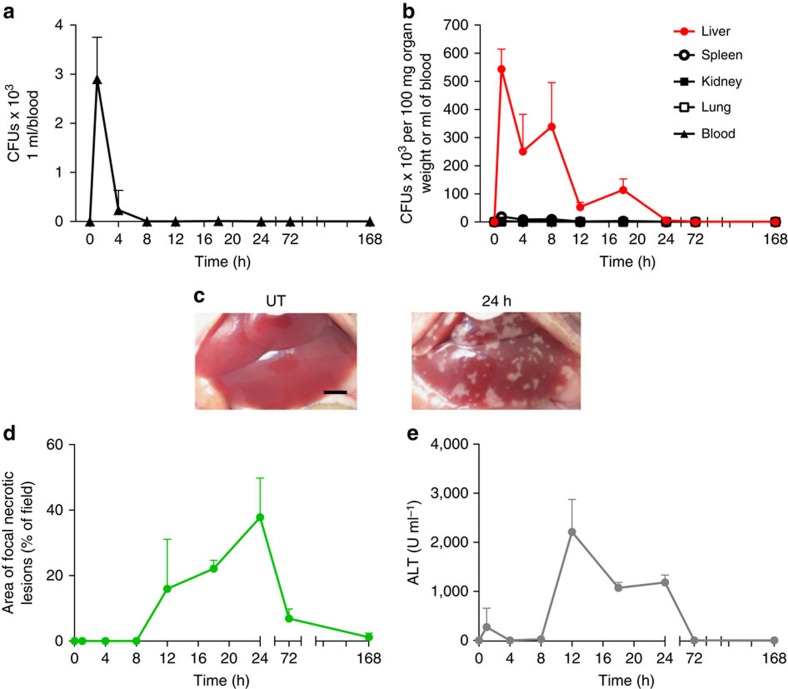
Systemic infection of mice with MRSA results in accumulation of bacteria in the liver and hepatic damage. Kinetic changes of the bacterial load in the blood (**a**), and all tested organs (liver, spleen, kidney and lung) (**b**) of methicillin-resistant *Staphylococcus aureus* (MRSA)-infected mice expressed as colony-forming units (CFUs). (**c**) Representative images of the healthy uninfected liver (untreated, UT), and liver collected from a mouse infected 24 h earlier with MRSA. The scale bar indicates 5 mm. (**d**) Changes in liver morphology evaluated by ImageJ as the area covered with focal necrotic loci, and (**e**) serum ALT levels were monitored in UT control (no infection) and septic mice over a period of 7 days. Data are shown as mean±s.d.; *n*≥3 per group.

Systemically injected MRSA was nearly completely cleared from the circulation within the first 4 h and not detected by 8 h (Fig. 1a). In investigated organs, including liver, kidney, lung and spleen (Fig. 1b, black lines and Supplementary Fig. 1a–c) MRSA colony-forming units (CFUs) could be detected mostly within the first 12 h. However, at all times investigated, the liver had 25–450 times higher CFUs than any other tissue (Fig. 1b red line). This significantly higher bacterial load was due to bacterial capture and sequestration by liver intravascular macrophages (Kupffer cells)^30^. Visualization of the liver for the first 30–60 s after MRSA injection reveals the tremendous ability of Kupffer cells, localized in the vasculature, to catch MRSA out of the blood stream in the first pass (Supplementary Movie 1). This was not dependent upon route of administration as injection via different vessels still resulted in the majority of MRSA in the liver.

MRSA induced significant pathological changes in the liver over the first 12–24 h post administration but rapidly resolved thereafter. Macroscopically, white areas that appeared to lack perfusion were noted on the surface of the liver (Fig. 1c,d), and they correlated with increased levels of alanine transaminase (ALT) indicative of hepatocyte necrosis (Fig. 1e). Further careful histopathological examination revealed numerous focal loci of necrotic hepatocytes, and infiltrating leukocytes (Supplementary Fig. 2a). The appearance of the necrotic loci upon MRSA treatment was observed in various strains of mice (C57Bl/6, Balb/c and B6.129) and has previously been reported in pigs and in humans^31^,^32^. Large liver abscesses are rare in humans^33^ and were not seen in our mouse model. In intravital experiments using FITC-albumin, these necrotic areas were found to be devoid of any blood flow while surrounding areas were well perfused (Supplementary Fig. 2b). In addition, MRSA infected mice exhibited sickness behaviour (apathy, lower locomotor activity) and loss of weight, and the latter did not fully recover even at 1 week post infection (Supplementary Fig. 3a). This dose of MRSA (1–2 × 10^7^) was sublethal but an increase of only 0.5–1 order of magnitude (5–10 × 10^7^) led to 40–50% mortality over the first 7 days (Supplementary Fig. 3b).

### MRSA-induced neutrophil recruitment mediates liver damage

**Figure 2:**
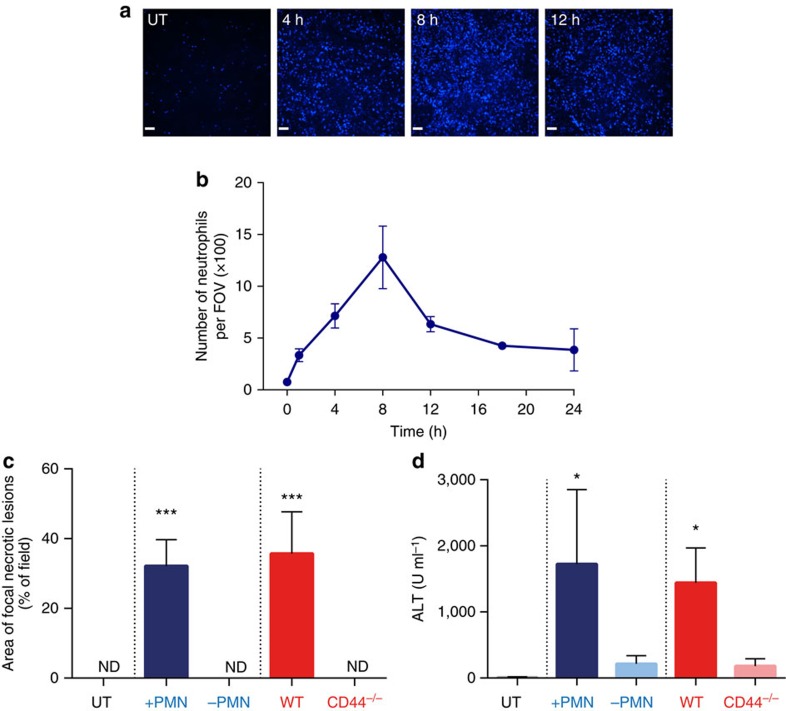
Neutrophils are indispensable for development of the damage to the liver. (**a**) Representative SD-IVM images of neutrophil infiltration into the liver sinusoids of MRSA-infected mice (magnification 4x). Images were taken just before bacteria injection (0 h/UT), and every few hours post MRSA inoculation. Neutrophils were stained with eFluor 660 anti-Gr-1 antibody (blue). Scale bar, 100 μm. (**b**) The numbers of infiltrating neutrophils as imaged in **a** were quantified with ImageJ software and are expressed as number per field of view (FOV). (**c**,**d**) Depletion of neutrophils with a high dose of neutrophil-depleting anti-Gr1 antibody (-PMN; blue bars) or lack of neutrophil recruitment in the liver of CD44^−/−^ mice (red bars; at 24 h of sepsis) unlike in their controls (+PMN) or wild-type controls (WT), respectively. (**c**) The area covered with altered hepatic tissue was evaluated with ImageJ, and (**d**) ALT levels were measured in serum. ND, not detected. *0.01<*P*≤0.05, ***0.0001<*P*≤0.001 (analysis of variance). Data are shown as mean±s.d.; *n*≥3 per group.

Intravital microscopy of healthy uninfected livers reveals that columns of hepatocytes (green autofluorescence) are perfused by liver sinusoids (black spaces between hepatocytes) with very few neutrophils (Ly6G^+^) present within the vessels (Supplementary Movie 2, part I). Following MRSA infection (at 24 h), architecture within this tissue was profoundly disrupted and numerous neutrophils could be seen crawling within the sinusoids in the vicinity of the necrotic lesions (Supplementary Movie 2, part II). A significant recruitment of neutrophils into the infected liver was observed as early as 1 h post infection and it continued over the next 8 h (Fig. 2a,b). Selective depletion of neutrophils before MRSA inoculation, completely abrogated the liver damage (Fig. 2c,d). A similar response was noted in CD44^−/−^ mice (Fig. 2c,d), which have normal numbers of circulating neutrophils that fail to be recruited to infected livers. This is because unlike in other organs, where neutrophil recruitment is selectin- and integrin-dependent, in the liver it relies on CD44 during systemic infection^34^.

### MRSA induces NET formation in the liver

**Figure 3:**
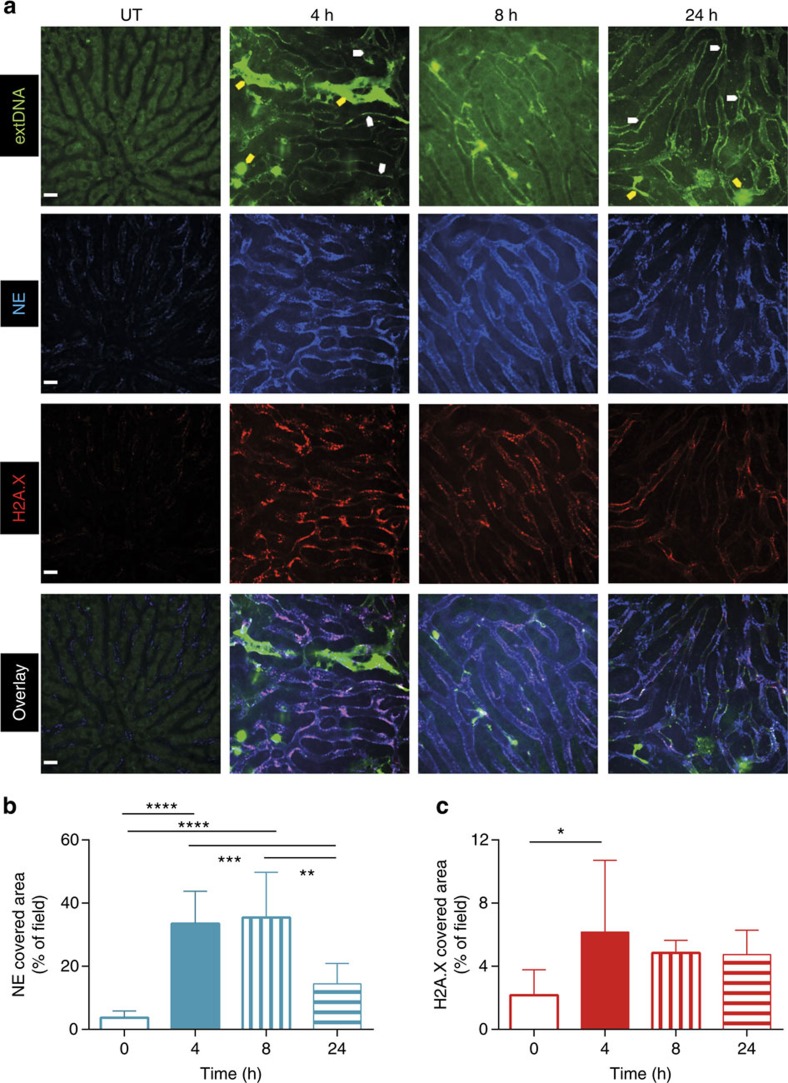
Neutrophils form NETs that persist for hours in liver vasculature during MRSA sepsis. (**a**) Representative images of NETs were acquired with SD-IVM in untreated (UT) mice, and animals injected with MRSA, 4, 8 and 24 h before imaging. Extracellular DNA (extDNA) was stained with Sytox Green (green^bright^), histones H2A.X with AF555-anti-H2A.X antibody (red) and neutrophil elastase (NE) with AF647-anti-NE antibody (blue). To visualize co-localization of NET components, the images from each channel were overlaid. Yellow arrows indicate Sytox^+^ necrotic cells, whereas white arrows denote (exemplary) DNA localization along sinusoid walls. The scale bar indicates 20 μm. Quantitative analysis of NETs within the livers of mice systemically infected with MRSA: (**b**) area of NE and (**c**) of H2A.X staining. Data are representative of three experiments. *0.01<*P*≤0.05, ^***^0.0001<*P*≤0.001 (*t*-test). Data are shown as mean±s.d.; *n*=3–10 per group.

To identify NETs using intravital microscopy, and to delineate these structures from free DNA released from damaged cells, we utilized a combination staining approach to visualize the co-localization of three cardinal NET components: extracellular DNA (extDNA), histones (H2A.X) and NE (Fig. 3a). In addition, MMP-9 was detected, and displayed the same staining pattern as NE (Supplementary Fig. 4a). extDNA was detected with cell impermeable Sytox green and could be seen lining the walls of the liver sinusoids (Fig. 3a). Histones and NE were clearly lining sinusoids suggesting NETs were anchored within the vasculature along the sinusoidal walls. In fact, all three structures clearly overlaid along the sinusoids. However, Fig. 3a (top row, green channel for extDNA) also reveals that at 4 h two Kupffer cells (top) and a few hepatocytes (lower left corner) took up Sytox green, which clearly did not overlap with antibodies for histones and NE (see overlay in Fig. 3a for 4 h) highlighting the importance of staining for all three structures to identify NETs. By 24 h, there were fewer Sytox-positive cells. Interestingly, at no time point did we see neutrophils take up Sytox green suggesting that they were neither permeable nor lysing (Supplementary Movie 3, magenta Ly6G^+^ neutrophils do not take up Sytox green). To confirm the identity of Sytox-positive structures, we used i.v. DNase to remove the bright green signal associated with DNA, which was lining the vasculature (Supplementary Movie 3). NET formation was most robust at 4 and 8 h and persisted beyond 24 h post infection (Fig. 3a–c), a time when bacteria were no longer detectable in the liver and circulation (Fig. 3a versus Fig. 1a,b).

**Figure 4:**
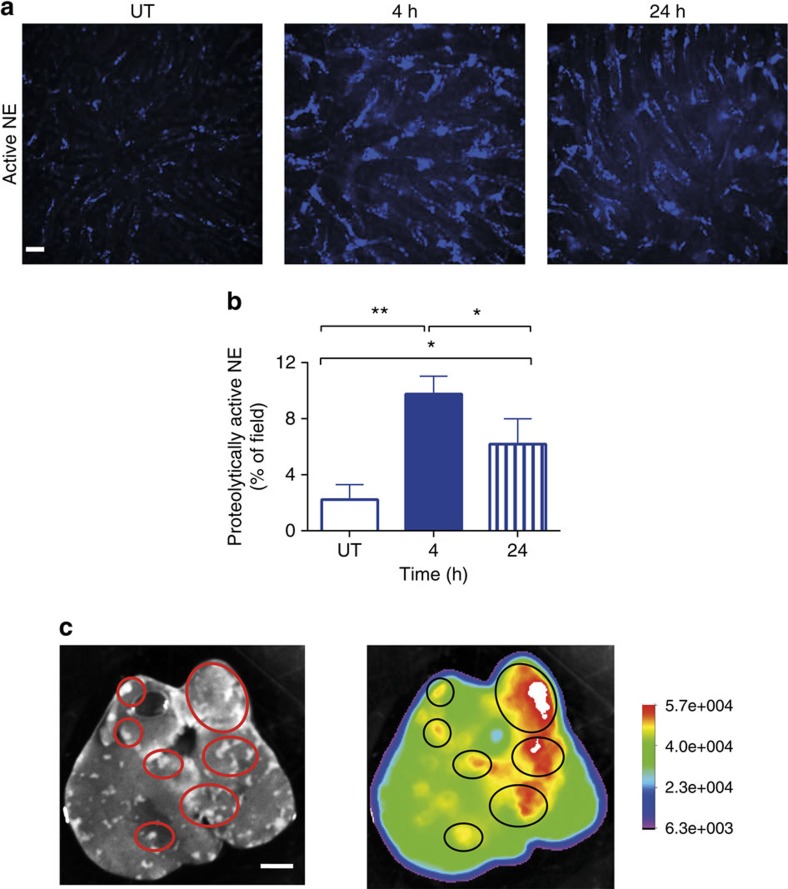
Neutrophil elastase attached to NETs is proteolytically active and co-localizes with necrotic areas. Activity of the enzyme was measured by *in vivo* zymography in which otherwise silent substrate becomes fluorescent in the presence of the active enzyme. (**a**) Representative images demonstrating the activation of the fluorescent substrate following successful processing by neutrophil elastase (NE), reflecting on its activity. The scale bar indicates 20 μm. (**b**) Quantitative analysis by ImageJ of NE activity within the livers of untreated (UT) mice, and animals infected with MRSA, 4 and 24 h before imaging. (**c**) Representative images of a liver extracted from a mouse inoculated with MRSA 24 h before imaging and injected with NE activity AF680-like probe. The black and white image reveals localization of necrotic areas on the liver surface, and the intensity rainbow image shows localization of the substrate effectively processed by active NE. Circles denote exemplary areas of co-localization of necrotic areas and active NE (as indicated by its processed substrate). The scale bar indicates 5 mm. *0.01<*P*≤0.05, **0.001<*P*≤0.01 (*t*-test). Data are shown as mean±s.d.; *n*=3 per group.

With a novel *in vivo* zymographic assay, we were able to determine that NE was enzymatically active at 4 h and remained active even 24 h post infection (Fig. 4a,b). In contrast, although MMP-9 protein was found in NETs, the activity of MMPs was very weak (Supplementary Fig. 4a versus b).

Intravital spinning disk microscopy revealed robust histones and NE staining in areas adjacent to the focal necrosis (Supplementary Fig. 5a). Interestingly, despite numerous attempts and various staining approaches we were not able to image within these necrotic areas. Therefore, an alternative approach, the highly sensitive *In-Vivo* Xtreme imaging instrument was used which measures the intensity of fluorescence emitted from whole organ explants. Using this approach, at 12 h post-infection, we identified clear patches of proteolytically active NE, which appeared to precede the formation of focal necrotic areas (Supplementary Fig. 5b). High levels of NE activity persisted in/nearby the necrotic areas at 24 h with enzymatic activity diminishing in areas located away from the lesions (Fig. 4c).

### DNase removes extracellular DNA but no other NET components

**Figure 5:**
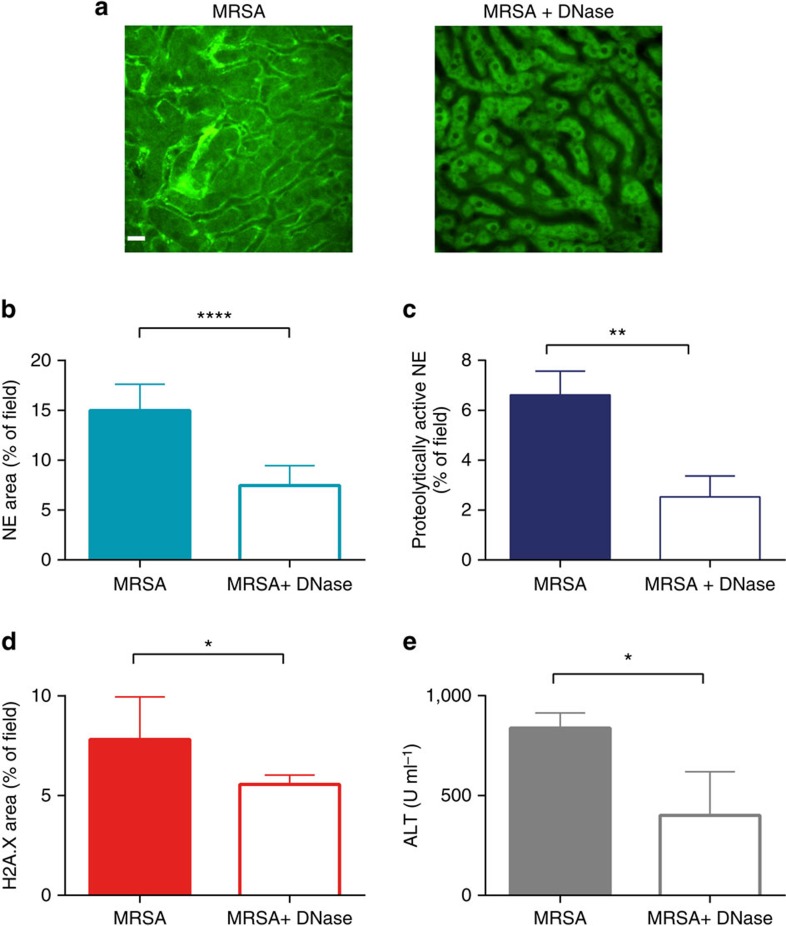
DNase efficiently removes extracellular DNA but only partially other NET components. DNase was applied intravenously 4 h after induction of MRSA sepsis (MRSA+DNase), and at 24 h post infection its effect on liver damage was compared with saline-treated mice (MRSA). (**a**) DNase treatment completely removed extracellular DNA (extDNA; representative images, scale 20 μm), but only partially removed other NET components: quantitative analysis of the NET area covered by (**b**) NE and (**d**) H2A.X staining 24 h after MRSA. (**c**) Quantitative analysis of NE activity at 24 h of sepsis as estimated by *in vivo* zymography detecting specifically NE processed fluorescent substrate (area covered by the substrate assessed with ImageJ). Changes in serum ALT levels were measured at 24 h (**e**). *0.01<*P*≤0.05, **0.001<*P*≤0.01, ****P<0.0001 (*t*-test). Data are shown as mean±s.d.; *n*=3–7 per group.

DNase was shown previously to breakdown NETs^6^,^12^, and *in vitro* to dissolve the whole NET structure^4^. Real-time observation, using spinning disk microscopy, revealed that the DNase immediately dissolves extDNA (1–2 min) without affecting histones and NE lining the walls of liver sinusoids (Supplementary Movie 4). When DNase was injected intravenously 4 h following infection with MRSA (after majority of NETs are formed), and then the livers were imaged at 24 h, again no extDNA was observed (Fig. 5a) but despite this, significant amounts of histones (70%) and NE remained (50%) attached to sinusoids (Fig. 5b,d). Furthermore, substantial NE proteolytic activity persisted (~50%; Fig. 5c), and so did the liver damage (Fig. 5e). The DNase treatment changed neither neutrophil infiltration (Supplementary Fig. 10a) nor bacterial clearance in this organ (Supplementary Fig. 11b). As an alternative to DNase treatment, heparin has been previously reported to mediate the *in vitro* dismantling of NET-associated DNA^16^. Also upon repeated high-dose heparin treatment, a significant amount of NE and histone signal persisted in liver sinusoids (Supplementary Fig. 6a,b), and some hepatic damage remained (Supplementary Fig. 6c,d).

### NET components anchor to VWF in vasculature

**Figure 6:**
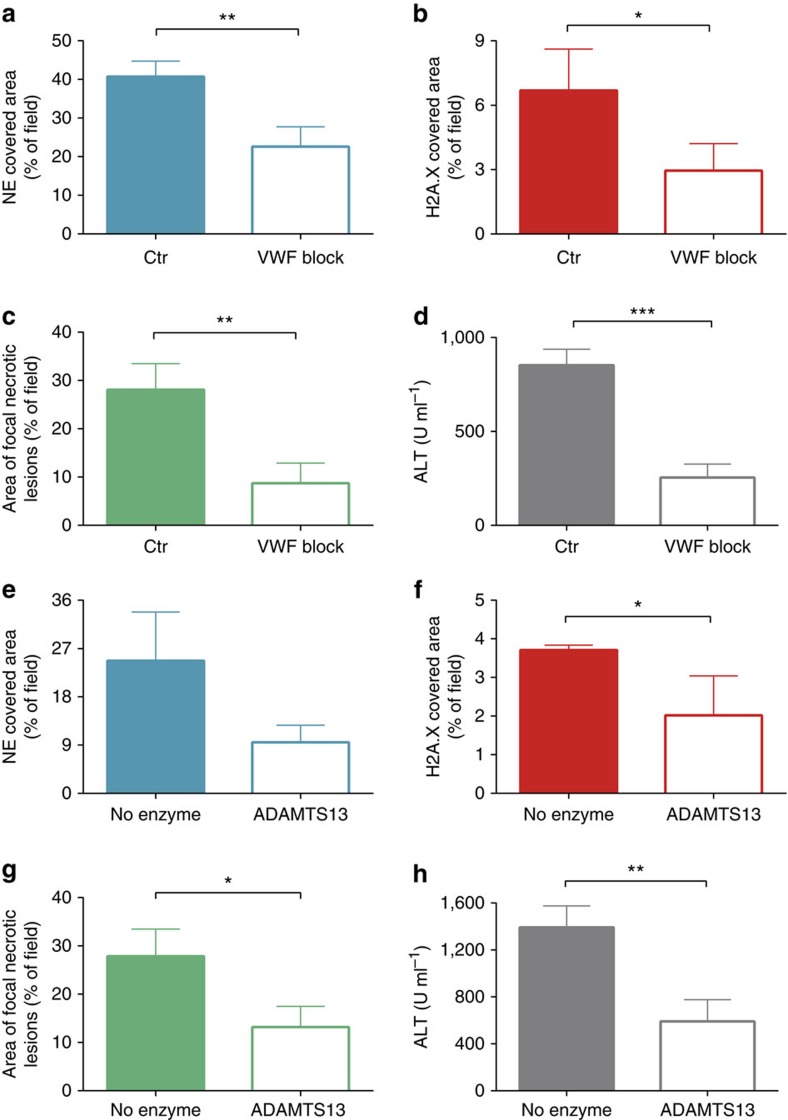
NET components attach extracellularly to von Willebrand factor. Von Willebrand factor (VWF) blocking antibody was applied i.v. 30 min before MRSA inoculation (VWF block) while control mice received rabbit serum (Ctr). Quantitative analysis of NETs within the liver sinusoids was performed at 4 h of sepsis: (**a**) area of NE and (**b**) H2A.X staining. At 24 h post MRSA inoculation, (**c**) area covered with necrotic tissue was assessed with ImageJ, and (**d**) serum ALT levels were measured. Alternatively, VWF was shed by ADAMTS13. In this scenario, mice were inoculated with MRSA and 8 h later they were injected intravenously with ADAMTS13. Sixteen hours later, their livers were imaged by SD-IVM to detect and quantify neutrophil elastase (**e**) and histones H2A.X (**f**). Impact of ADAMTS13 on liver damage as estimated by area covered with necrotic tissue (**g**) and ALT levels (**h**). *0.01<*P*≤0.05, **0.001<*P*≤0.01, ***0.0001<*P*≤0.001 (*t*-test). Data are shown as mean±s.d.; *n*≥3 per group.

Following the unexpected observation that not all NET components detach upon DNase treatment, we aimed to identify vasculature lining molecule(s) to which the components bind. We have previously shown that under both basal conditions and during systemic MRSA infection, VWF lines the liver sinusoids and Kupffer cells^30^. Furthermore, this molecule, normally associated with hemostasis, has been shown to bind histones^35^ raising the possibility that VWF mediates anchoring of NETs within the liver vasculature. Pre-treatment of mice with VWF blocking antibody did not affect neutrophil recruitment to the liver (Supplementary Fig. 10f) but reduced the histone binding by 50% and reduced liver damage by nearly 80% (Fig. 6b, c–d, respectively). In addition, blocking VWF prevented the accumulation of DNA (Supplementary Movie 5) within the liver vasculature and significantly reduced (by half) the amount of the elastase within the sinusoids (Fig. 6a and Supplementary Movie 5), suggesting that VWF anchors the entire NET structure to the blood vessel walls. To further verify these results, we i.v. administered ADAMTS13, a protease that removes VWF from the vessel wall. ADAMTS13 was applied at 8 h so it could not, and did not (Supplementary Fig. 10e), affect neutrophil recruitment. ADAMTS13 reduced VWF-dependent NET adherence to the vascular wall (Fig. 6e,f), and resulted in diminished hepatic damage (Fig. 6g,h).

### Prevention of NET release eliminates liver damage

The specific mechanisms leading to *in vivo* NET production remain controversial. Oxidants have previously been purported to induce NET formation *in vitro* in response to phorbol myristate acetate (PMA)^16^. NET formation and liver injury following MRSA infection of Ncf^−/−^ mice (deficient for p47^phox^ in myeloid cells, thus not generating reactive oxygen species (ROS) by these cells) or of animals treated with apocynin (general nicotinamide adenine dinucleotide phosphate (NADPH) inhibitor) was similar to that observed in wild-type mice (Supplementary Fig. 7a–d). Thus, *in vivo* NET production in response to systemic MRSA infection does not depend on NADPH oxidase-dependent oxidants. Although oxidant production from other sources cannot be excluded, the source was not NADPH oxidase.

**Figure 7:**
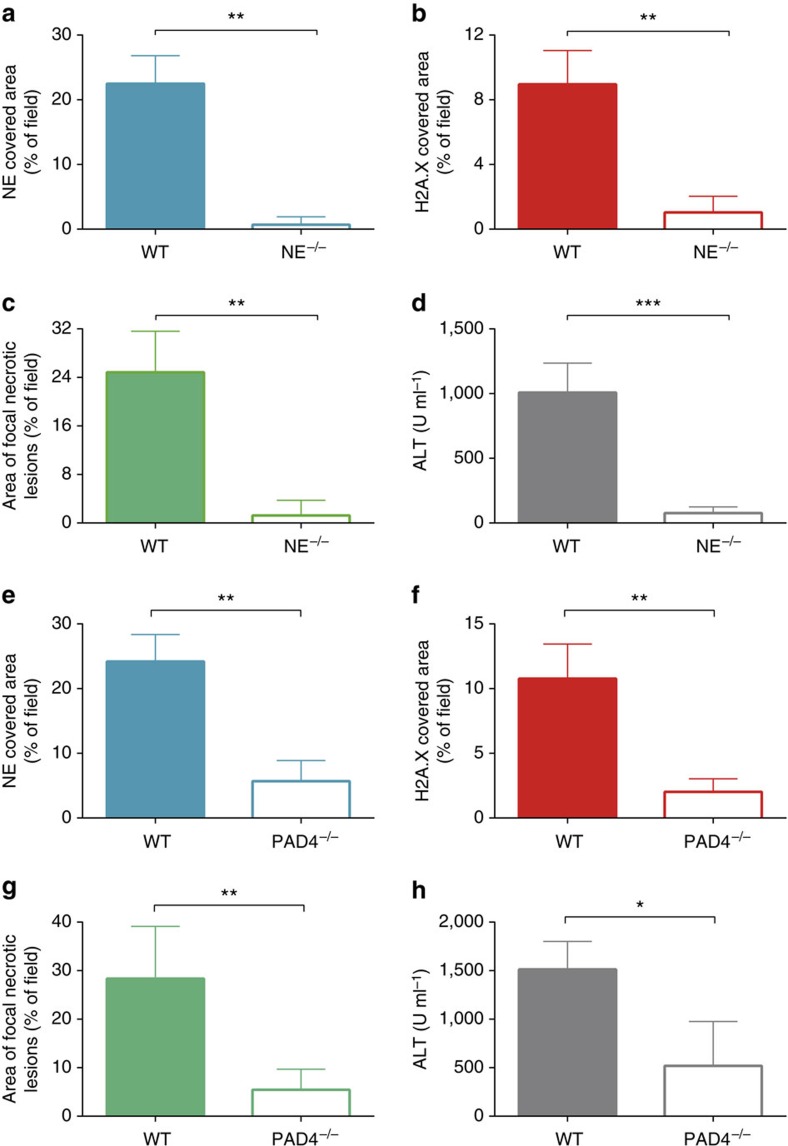
Impaired release of NETs by NE- and PAD4-deficient mice during MRSA sepsis. Quantitative analysis of NETs within the livers of neutrophil elastase-deficient (NE^−/−^) mice and their wild-type controls (WT) systemically infected with MRSA for 4 h: (**a**) area of NE and (**b**) H2A.X staining. Hepatic damage was evaluated at 24 h post MRSA inoculation: (**c**) area covered with altered tissue was assessed in NE^−/−^ and WT mice with ImageJ, and (**d**) ALT levels were measured in serum. Quantitative analysis of the NET area covered by NE (**e**) and (**f**) H2A.X staining at 4 h after MRSA infection in PAD4^−/−^ and WT mice. (**g**) Evaluation of the liver area with altered morphology and (**h**) serum levels of ALT in PAD4^−/−^ and WT mice at 24 h post MRSA inoculation. *0.01<*P*≤0.05, **0.001<*P*≤0.01, ***0.0001<*P*≤0.001 (*t*-test). Data are shown as mean±s.d.; *n*=3–5 per group.

**Figure 8:**
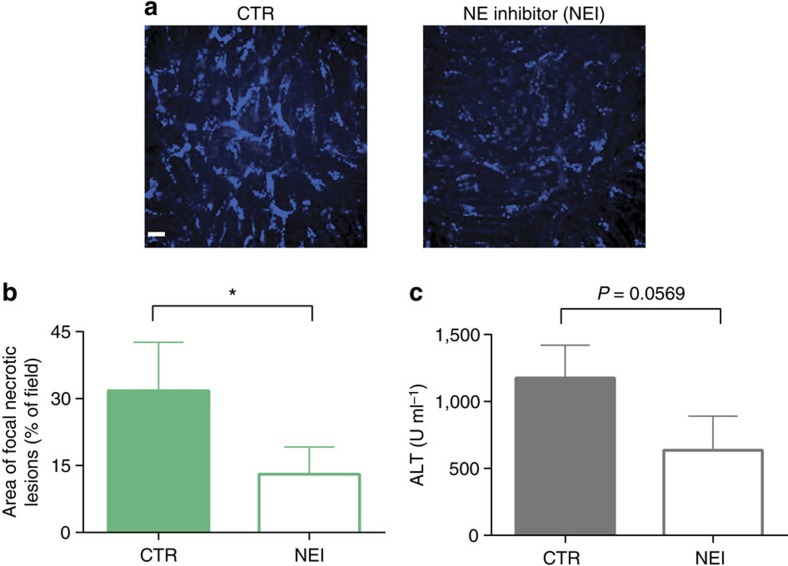
Inhibition of neutrophil elastase activity diminishes MRSA-triggered damage to the liver. Inhibitor of NE was applied after NETs were formed and released to vasculature. (**a**) Representative images of liver sinusoids laid with substrate processed by NE (AF680-like probe) in MRSA-treated mice (CTR) and animals treated with NE inhibitor sivelestat (NEI) at 4 h of sepsis. The scale bar indicates 20 μm. (**b**) Changes in liver morphology evaluated 24 h post MRSA inoculation by ImageJ and (**c**) serum ALT levels estimated in mice treated with either NE inhibitor (NEI) or saline (CTR). *0.01<*P*≤0.05 (*t*-test). Data are shown as mean±s.d.; *n*≥3 per group.

Elastase has been previously shown to be critical for histone degradation leading to DNA decondensation and NET formation^9^. MRSA infection of NE-deficient mice was by far the most effective way of reducing NET production (Fig. 7a,b) and liver damage (Fig. 7c,d). When an elastase inhibitor was administered 4 h following MRSA infection, NETs still formed during the first 4 h but the subsequent protease activity of NE was reduced (Fig. 8a). This approach attenuated damage to the liver by approximately 50% (Fig. 8b,c). NE could be released either through degranulation or as a portion of NETs. In a final series of experiments, we used an NE independent way of preventing NET production, namely in PAD4-deficient mice^8^. PAD4 deficiency dramatically reduced NET release following MRSA infection (Fig. 7e,f). Moreover, PAD4-deficient mice demonstrated significantly reduced liver damage (~80%; Fig. 7g,h) suggesting that NET proteolytic activity (based on NE^−/−^ data) accounts for about 80% while the remaining 20% might be related to elastase release via degranulation.

Deficiency in the other NET-associated protease studied here, namely MMP-9, resulted in no inhibition of NET formation and no inhibition of liver damage (Supplementary Fig. 8a–d). Moreover, the remaining hepatic injury in PAD4^−/−^ mice was not caused by ROS themselves (unrelated to NETs) as NADPH inhibitor did not further limit the damage in PAD4-deficient mice (Supplementary Fig. 9a,b). Finally, we addressed the very important issue of whether the damage could be caused by *S.aureus* toxins. There are more than ten toxins produced by *S.aureus* including three bicomponent leukocidins, Luk AB/GH, Panton Valentine Leukocidin, γ-haemolysin, the pore-forming α-haemolysin, δ-toxin and phenol-soluble modulins (PSM) α^36^. The *agr* operon constitutes a global regulatory system (the master switch) that controls cell density-dependent expression of majority of *S. aureus* virulence factors^38^. We used a mutant MRSA that is lacking *agr* operon that results in an *S. aureus* that expresses none of the aforementioned toxins^37^. When we used this mutant we still saw ample NET production and liver damage (Supplementary Fig. 12).

## Discussion

Methicillin-resistant *S. aureus* (MRSA) is one of the most common blood isolates during sepsis^27^ and indwelling intravenous catheters are among the most common reasons for MRSA infection^39^. Community-associated (CA)-USA300 is the dominant CA strain and represents the vast majority of *S. aureus* infections in the North America^40^. Although most of the superficial skin infections are still readily managed with antibiotics, when bacteremia is associated with skin infections, the results are far more devastating, resulting in up to 30% mortality^26^,^39^. There is growing evidence that sepsis-associated deaths are not due to the microorganism *per se*, but rather are due to the persistent activation of innate immunity, resulting in intractable inflammation and organ injury^28^,^41^. Our model of MRSA bacteremia in mice in part corroborates this theory, but in part provides novel information. We report herein that upon entry of *S.aureus* into the vasculature, the reticuloendothelial system and more specifically, the Kupffer cells of the liver, very rapidly sequester the majority of the *S.aureus*. This leads to a transient increase in liver enzymes, and some liver necrosis that appears to be entirely the result of neutrophil recruitment, activation and NET release and that this damage was eliminated by 80% if neutrophil-mediated NET production (PAD4 deficiency) was blocked.

It is worth noting that the liver enzymes quickly returned back towards control levels and the liver repaired rapidly in our mouse model and overt abscesses were not noted. In humans, MRSA-induced liver abscesses are also not commonly described^33^, perhaps due to the profound and rapid healing power of the liver. However, some liver necrosis is not unusual, and in humans if microabscesses develop they are potentially life-threatening^31^,^42^, and multiple liver abscesses lead to higher mortality than single abscesses^33^. The elevated liver enzyme levels, and even the observed liver lesions in our model, only lasted 24–72 h and would likely have been missed in humans who are often infected for prolonged time points before entering intensive care units. Nevertheless, during MRSA bacteremia, hepatic damage (increased ALT) is regularly reported^43^ and similar areas of focal necrosis with neutrophil infiltration and vascular leakiness were also reported in other model species such as pigs^32^.

Liver resident macrophages (Kupffer cells) provide the first defence to many disseminating pathogens that enter the bloodstream^44^. We have recently reported that immediately upon bacterial capture by Kupffer cells, and well before any neutrophils arrive, platelets that appear to patrol the liver by a touch-and-go interaction with Kupffer cells, swarm and encapsulate caught *S. aureus*, helping to eradicate the infection^30^. Ironically, this interaction is dependent upon VWF, the very same molecule that localizes NETs to the vasculature at later time points and leads to significant liver damage. We used lower concentrations of *S.aureus* than in our previous study^30^ to ensure that mice survived the early essential need for VWF. It is possible that at this lower concentration the VWF and platelets are not needed to help eradicate infection. This is another example of a molecule (in this case VWF) that is essential to help the host survive the early phase of an infection, but with time the molecule is no longer of benefit and in this case causes some bystander injury via NET anchoring to the vessel wall. The neutrophils are likely summoned by the Kupffer cells, and upon arrival and activation in the liver, start rapidly making NETs that anchor via VWF and persist for at least 24 h.

Our *in vivo* data revealed that NET-associated NE is proteolytically active within the vasculature. This result is particularly interesting as it demonstrates that even in the presence of a high concentration of endogenous protease inhibitors (10% of plasma proteins)^45^,^46^, this critical protease remains active. The possibility that DNA can protect active proteases was previously reported in simple *in vitro* studies utilizing co-incubation of elastase and DNA^23^,^24^, the observation described well before NETs were discovered. Our findings with MRSA-induced intravascular NETs place these previous results in a new context and underscore the potential significance of that work^23^,^24^. However, it is important to note that not all NET-bound proteases were observed to be active. The low MMP activity associated with intravascular NETs, despite strong deposition of MMP-9 protein, might be explained by the fact that NE is stored in neutrophilic granules as the active protease, whereas MMP-9 is released as zymogen requiring extracellular activation^47^,^48^.

Blocking the production of NETs (NE^−/−^ and PAD4^−/−^ phenotypes) almost completely protected the liver from MRSA-induced damage, suggesting that it is the NET (and associated components), and not the pathogen, that is entirely responsible for the observed damage. The potential for NE to induce significant bystander tissue destruction has been recognized for decades^49^ and our data suggest that all of the liver injury is attributable to NE and a large portion (at least 80%) of that damage is attributable to NET production as PAD4^−/−^ mice, which could not make NETs, but could release elastase through degranulation, had an 80% reduction in liver injury. Consistent with this conclusion is the slightly higher deposition of NE in the liver vasculature of PAD4^−/−^ mice than in NE^−/−^ animals and this could presumably be through degranulation. Histones have also been shown to induce direct injury to endothelium in sepsis^19^, and although these molecules did not prove to be the dominant cytotoxic molecule in our model, the role of histones as a potential anchor protein for the entire NET structure should not be underestimated. Indeed, blocking VWF dislodged each of NET components from the liver vasculature and reduced tissue injury by 80%. It is also worth mentioning that the histones may specifically target endothelium^19^, whereas we measured liver enzymes that are more reflective of hepatocyte necrosis.

DNase efficiently degrades extracellular DNA and is successfully used to diminish symptoms of cystic fibrosis^13^. Although this potential therapy may be tempting in sepsis, our data suggest that DNase fails to clear all of the NET components, as histones and elastase remain bound to the endothelium in sufficient amounts to cause significant liver injury, even in the presence of DNase.

Almost 20 years ago, Ward and colleagues identified an interesting potent biochemical interaction between histones and VWF of unknown physiological role^35^. And recently, VWF was found to directly interact with DNA of NETs^50^ and co-localize with them^16^. Here we have identified VWF as a critical anchor for NETs within the liver vasculature, and that disruption of this interaction not only reduced adherence of histones and elastase to the sinusoidal endothelium but also reduced DNA binding to the vessel wall, suggesting that the entire NET structure was disrupted. What remains unclear is the specific nature of this interaction. A prevailing view is that similar to NET binding to bacteria, the interactions between histones and VWF, are based on electrostatic forces, mediated by positively charged amino acids within the protein binding to anionic DNA^4^,^51^. This would also explain how the infusion of non-adhering heparin, carrying the strongest negative charge of all biological polymers^52^, was able to destabilized the NET structure *in vivo*. In fact, a recent *in vitro* study showed that when incubated together, DNA, histones and heparin formed complexes and that when the heparin concentration increased, DNA was displaced, and the histone-heparin dimers dominated^53^. Also NE was shown to be sensitive to electrostatic interactions as it can be sequestered by negatively charged gauze (used for wound healing) via its positively charged side chain residues^54^.

In summary, this report highlights that NETs adhere to the vessel wall, persist for at least 24 h, and, importantly, retain much of their proteolytic activity. DNase, despite being highly efficient at removing DNA, is much less effective at removing some of the most toxic components of NETs. The persistence of NET-associated proteases within the liver vasculature, even after the dissolution of the NET structure, suggests that strategies that either inhibit NET production or induce the shedding of anchor molecules, such as VWF, might offer greatest efficacy in preventing NE-associated tissue damage.

## Methods

### Mice

Animal experiments were carried out with male adult mice (7- to 10-week old) and all experimental animal protocols were approved by the University of Calgary Animal Care Committee and were in compliance with the Canadian Council for Animal Care Guidelines. The C57Bl/6J mice, NE-deficient mice (NE^−/−^), B6(Cg)-Ncf1m1J/J mice (Ncf^−/−^) were purchased from Jackson Laboratories. MMP-9-deficient mice were generated at the Rega Institute for Medical Research, University of Leuven (Belgium)^47^. Peptidylarginine deiminase 4-deficient mice (PAD4^−/−^), mice were a kind gift from Dr Kerri Mowen at the Scripps Research Institute (La Jolla, CA, USA). All of the above mice were generated on the C57Bl/6J background. In addition, CD44-deficient mice (CD44^−/−^) and their respective WT strain (B6.129) were also purchased from Jackson Laboratories. All animals were maintained in a specific pathogen-free environment at the University of Calgary Animal Resource Centre. Mice were housed under standardized conditions of temperature (21–22 °C) and illumination (12 h light/12 h darkness) with free access to tap water and pelleted food. Changes in animal weight were monitored at the same time daily (morning).

### Materials and treatments

Purified goat anti-mouse histone H2A.X (M20) and goat anti-mouse NE (M18) and goat anti-mouse MMP-9 (M17) antibodies were purchased from Santa Cruz Biotechnology (catalogue no. sc-54607, sc-9521, sc-6841, respectively). For intravital microscopy, NE and histone H2A.X antibodies were conjugated to Alexa Fluor 647 or Alexa Fluor 555 with protein labelling kits as per the manufacturer’s instructions (Invitrogen). Labelling of neutrophils was performed using either Alexa Fluor 750-conjugated anti-Ly6G (1A8) from AbLab (custom made) or eFluor 660-conjugated anti-Gr-1 (RB6-8C5) from eBioscience (catalogue no. 50–5931). Kupffer cells were stained with PE-anti-F4/80 (BM8; eBioscience; catalogue no. 12–4801). Sytox green DNA dye was purchased from Invitrogen. For the degradation of intravascular DNA, 2,000 U of cell culture grade DNase I (Roche) were administered i.v. at the time of bacterial injection or 4 h post infection. In some experiments, the 2,000 U of DNase i.v. were used to remove extDNA in real-time during imaging.

For detection of active proteases, FAST Fluorescent Imaging Agents (PerkinElmer) were used: Neutrophil Elastase 680 and MMPSense 750. The optically silent compounds become fluorescent following specific protease-mediated activation. These assays were confirmed for selectivity using both knockout mice and inhibitors.

Two NE inhibitors were used, elastase inhibitor IV (Calbiochem/EMD Millipore) and sivelestat (Sigma-Aldrich). The elastase inhibitor IV was administered i.p. at the dose of 50 mg kg^−1^ and sivelestat was injected i.v. at the dose of 10 mg kg^−1^. Both inhibitors were administered at 4 h and again at 12 h after MRSA injection^55^ and yield similar results; data for sivelestat are shown in figures. NADPH inhibitor apocynin (acetovanillone; Sigma-Aldrich) was delivered i.v. at the dose of 10 mg kg^−1^, 30 min before MRSA injection^56^,^57^. Unfractionated heparin (Sandoz, Boucherville, Canada) and low-molecular-weight heparin Fragmin anti-factor Xa (Pfizer) were administered s.c. at 400 U kg^−1^. Heparins were administrated at time 0 and again at 12 h after MRSA injection. Data for unfractionated heparin are shown on figures.

Neutrophil depletion was performed by i.p. injection of 200 μg unconjugated anti-Gr-1 antibody (1A8; BioXcell; catalogue no. BE0075-1) 48 and 24 h before bacterial injection. Control mice were injected with matching rat IgG2b antibodies (BE0089).

### Bacterial strain and bacterial treatments

Clinically isolated CA-MRSA (US300-2406) carrying chloramphenicol resistance genes^58^ was used in the study. In addition, USA300 LAC (unlabelled or GFP) and USA300 LACΔAgr mutant were studied. MRSA was grown in BHI (Difco Brain Heart Infusion, BD) supplemented with chloramphenicol (20 μg ml^−1^, Calbiochem) on an orbital shaker at 37 °C overnight. Subsequently, bacteria were subcultured in fresh BHI and incubated for 2 h at 37 °C with shaking to obtain bacteria in mid-log phase growth before injection. Bacteria were washed, suspended in saline and mice were injected via the tail vein with 1–2 × 10^7^ CFUs in a volume of 200 μl per mouse. In some experiments, mice were injected with 5–10 × 10^7^ of CFUs.

### Evaluation of the size of damaged areas on liver surface

At specified time points post MRSA injection, the livers of euthanized mice were removed and immediately placed in ice-cold PBS. Images of the livers were captured with a digital camera and the affected areas on the surface of each liver were quantified with ImageJ (NIH).

### Perfusion of the liver

To determine perfusion of the liver microvasculature, FITC-labelled albumin (5 mg ml^−1^, 50 μl per mouse; Sigma-Aldrich) was i.v. administered into an anaesthetized mouse undergoing intravital imaging. Videos were recorded for 10 min to visualize appearance of FITC-albumin in the vasculature. The perfused sinusoids turned green immediately and the unperfused sinusoids remained unlabelled during the 10-min period.

### Biochemical assessment of liver injury

Anaesthetized mice were washed with 70% ethanol under sterile conditions. Blood was collected by cardiac puncture in a heparinized syringe. Samples were centrifuge at 400*g* for 10 min for the retrieval of plasma. Plasma samples were analysed for ALT levels as per manufacturer’s protocol (Biotron Diagnostics Inc.).

### Liver histology

The left liver lobe was removed and placed in 10% neutral-buffered formalin (Sigma-Aldrich). The formalin-fixed tissue was embedded in paraffin, cut in 4-μm sections and stained with haematoxylin and eosin by Calgary Laboratory Services.

### Bacteriological analysis

Anaesthetized mice were washed with 70% ethanol under sterile conditions. Blood was collected by cardiac puncture. Subsequently, mice were euthanized and the lungs, liver, right kidney and spleen were removed and homogenized. For determination of CFUs, 10 μl of tissue homogenate or blood was serially diluted, plated onto BHI agar plates (Difco Brain Heart Infusion Agar, BD) supplemented with chloramphenicol (20 μg ml^−1^) and incubated at 37 °C for 24 h followed by colony enumeration.

### Preparation of the mouse liver for intravital microscopy

Mice were anaesthetized with a mixture of ketamine hydrochloride (200 mg kg^−1^, Bayer Inc. Animal Health) and xylazine hydrochloride (10 mg kg^−1^, Bimeda-MTC, Cambridge, Canada). After anaesthesia, cannulation of the right jugular vein was performed for maintenance of anaesthetic and for injection of antibodies or other reagents. Preparation of the liver for intravital imaging was performed as previously described^59^. Briefly, a midline incision followed by a lateral incision along the costal margin to the midaxillary line was performed to expose the liver. The mouse was placed in a right lateral position, and ligaments attaching the liver to the diaphragm and the stomach were cut, thus allowing the liver to be externalized onto a glass coverslip located on the inverted microscope heat-controlled stage with all blood flow remaining intact. The liver was draped with a saline-soaked KimWipes to avoid tissue dehydration and to help restrict movement of the tissue on the slide.

### Spinning disk confocal intravital microscopy

The exposed liver lobe was visualized with an Olympus IX81 inverted microscope (Olympus) equipped with a confocal light path (Wave-Fx; Quorum) based on a modified Yokogawa CSU-X1 head (Yokogawa Electric Corporation) with a UPLANSAPO 10 × /0.40 or UPLANSAPO 20 × /0.70 air objective. Four laser excitation wavelengths (491, 561, 643 and 730 nm; Cobalt) were used in rapid succession and visualized with the appropriate long-pass filters (Semrock and Chroma). Exposure times for excitation wavelengths were 400 ms for all lasers. A back-thinned EMCCD 512 × 512 pixel camera (C9100-13, Hamamatsu) was used for fluorescence detection. Volocity acquisition software (PerkinElmer) was used to drive the microscope.

### Analysis of spinning disk confocal microscope-acquired images

Fluorescence imaging of NET components and neutrophil counts was performed with intravital immunofluorescence analysis. Neutrophils were visualized by injection of Alexa Fluor-conjugated 750 anti-mouse Ly6G (3 μg) or eFluor 660-conjugated anti-Gr-1 (1.6 μg) antibodies. Extracellular DNA was labelled with Sytox Green DNA dye (5 μM), histone H2A.X was labelled with Alexa Fluor 555-conjugated anti-mouse H2A.X antibody (5 μg) and NE was labelled with either Alexa Fluor 647-conjugated or Alexa Fluor 555-conjugated anti-mouse NE antibody (0.6 μg). Alexa Fluor 647-conjugated anti-mouse MMP-9 antibody (0.6 μg) was used for detection of MMP-9. Kupffer cells were stained with PE-anti-F4/80 (2.5 μg). All antibodies and dyes were injected i.v. 15 min before intravital imaging.

Neutrophils and NETs were quantified with SD-IVM using previously published methodology^59^. In brief, images were acquired as *z* stacks of *xy* planes (1 μm intervals) from the bottom to top of sinusoids in each field of view using a × 20 objective lens, and saved as extended focus images in.tiff format. Images from individual colour channels (for example, red for histone H2A.X, far red for elastase) were exported and analysed in ImageJ (NIH). Neutrophils were counted per 10x field of view (FOV), minimum 4 FOV from each mouse. Intensity of histone and elastase staining, and of protease processed substrate, was analysed so that differences in background fluorescence between experiments and antibody lots could be accounted for and background autofluorescence could be eliminated, contrast was adjusted to minimize autofluorescent background staining, and a minimum brightness threshold was set to yield only positive staining. The same contrast and threshold values were applied to all images from all treatment groups within the experiment. Thresholded images were converted to binary (black and white), and the area per field of view covered by positive fluorescence staining (black) was calculated with ImageJ software. Data are expressed as the percentage of area in each FOV covered by positive fluorescence staining.

### NE activity in the whole liver

Optical and X-ray small animal imaging system (In-Vivo Xtreme, Bruker) was used to quantify, and localize active elastase within the liver. Live mid-log phase *S. aureus* were administered i.v. together with Neutrophil Elastase 680 FAST Fluorescent Imaging Agents (PerkinElmer). At either 12 or 24 h post infection, livers were removed and placed in the imaging chamber. Livers collected from untreated (UT) mice were used as controls. Photons were measured during a 2-s exposure. Total photon emissions from uniform area of each mouse liver were quantified by using the Molecular Imaging (MI) software (Bruker).

### Studies on NET component attachment to VWF

For inhibition of VWF, each mouse was given intravenous injection of 50 μg polyclonal rabbit antibody to human VWF with strong cross-reaction with mouse VWF (Dako; catalogue no. A0082) 30 min before infection^30^. Normal rabbit serum (Sigma-Aldrich) was used as a control. ADAMTS13 (3 μg per mouse; R&D) was also used in some experiments. In pilot experiments, recombinant ADAMTS13 that starts at the N-terminus of the pro-domain and ends in the spacer domain was tested against the full-length molecule for its efficiency to shed VWF. No significant differences were recorded and the shorter rhADAMTS13 was used in subsequent studies. MRSA sepsis was induced and 8 h later ADAMTS13 was injected into the tail vein. This time point was selected not to influence neutrophil recruitment that reaches its maximum 8 h post MRSA. Liver sinusoids were imaged 16 h later (at 24 h) for NET components, and damage to the organ was assessed as described above.

### Statistics

All data are presented as mean values±s.d. Data were compared either by unpaired two-tailed Student’s *t*-test or one-way analysis of variance with Bonferroni multiple comparisons *post hoc* test. Statistical significance was set at *P*<0.05. Sample size was determined on the basis of prior power calculations and our experiments applying intravital microscopy^58^,^59^.

## Author contributions

E.K. designed and performed the experiments, collected, analysed and discussed data and wrote the manuscript. B.G.S. did the skin imaging. C.N.J., W.-Y.L., M.-J.S. performed some experiments and discussed the data. A.T. assisted the lung imaging. K.M. provided PAD4-deficient mice. G.O. provided MMP-9-deficient mice and discussed results. P.K. co-designed experiments, supervised the study and co-wrote the manuscript.

## Additional information

**How to cite this article**: Kolaczkowska, E. *et al*. Molecular mechanisms of NET formation and degradation revealed by intravital imaging in the liver vasculature. *Nat. Commun*. 6:6673 doi: 10.1038/ncomms7673 (2015).

## Supplementary Material

Supplementary Information Supplementary Figures 1-12

Supplementary Movie 1 Catching of MRSA by Kupffer cells in the liver. Liver of naïve mouse was prepared for intravital imaging. Kupffer cells were stained with Alexa-555-anti-F4/80 antibody (red). One minute after recording of a video was initiated, GFP-labelled MRSA was injected. The video was recorded for app. 10 minutes. Majority of GFP-MRSA was immediately engulfed by Kupffer cells as seen by the change of the green signal into the yellow one. The scale bar indicates 30 μm.

Supplementary Movie 2 Changes to liver architecture: morphology of healthy liver imaged with SD-IVM (part I) and liver of a mouse infected with MRSA twenty-four hours prior to imaging (part II). Neutrophils were labeled with AF750-anti-Ly-6G antibody (magenta). Autofluorescent hepatocytes are green. The scale bar indicates 20 μm. Each video was recorded for approximately 10 minutes, for details see the time stamps

Supplementary Movie 3 Staining for extracellular DNA (extDNA) and its removal by DNase. Intravascular DNA was labeled with Sytox green (shown by green fluorescence^bright^) and neutrophils with AF750-anti-Ly6G antibody (magenta). At the indicated time point, 2000 U DNase was infused i.v., and the digestion of extDNA could be observed by the loss of intravascular Sytox Green^bright^ staining. The scale bar indicates 20 μm. The video was recorded for approximately 13 minutes, for details see the time stamp.

Supplementary Movie 4 DNase is efficiently removing extDNA but not neutrophil elastase (NE) or histones (H2A.X). ExtDNA is shown in bright green (Sytox Green), histone H2A.X in red (AF555-anti-H2A.X antibody), and neutrophil elastase in blue (AF647-anti-NE antibody). All channels (green-blue-red) were recorded at the same time but are shown here separately for clarity. Part I: removal of extDNA, part II: the effect of DNase on NE, part III: the impact of DNase on H2A.X. The scale bars indicate 20 μm. The video was recorded for approximately 10 minutes, for details see the time stamp.

Supplementary Movie 5 Blockage of Won Willebrand factor (VWF) reduces attachment of extDNA and other NET components, such as neutrophil elastase (NE), to liver vasculature. Mouse A was injected only with MRSA and imaged 24 hour later. Mouse B was first injected with anti-VWF blocking antibody, 30 minutes later with MRSA, and then imaged after 24 hours. At the time of imaging, AF647-anti-NE antibody was injected via the jugular vein to stain neutrophil elastase (blue), and Sytox Green was infused by the same route to stain extDNA in real time (bright green). For each mouse, both channels (green and blue) were recorded at the same time but are shown here separately for clarity. Part I: staining of extDNA, part II: staining of NE. The scale bars indicate 20 μm. The video was recorded for approximately 10 minutes, for details see the time stamp.
