# Supplementary material for: Molecular mechanisms of NET formation and degradation revealed by intravital imaging in the liver vasculature
Source: Nat Commun. 2015 Mar 26;6:6673. doi: 10.1038/ncomms7673 (PMC4389265; doi:10.1038/ncomms7673)
Supplement: Supplementary Information — Supplementary Figures 1-12 [file ncomms7673-s1.pdf]

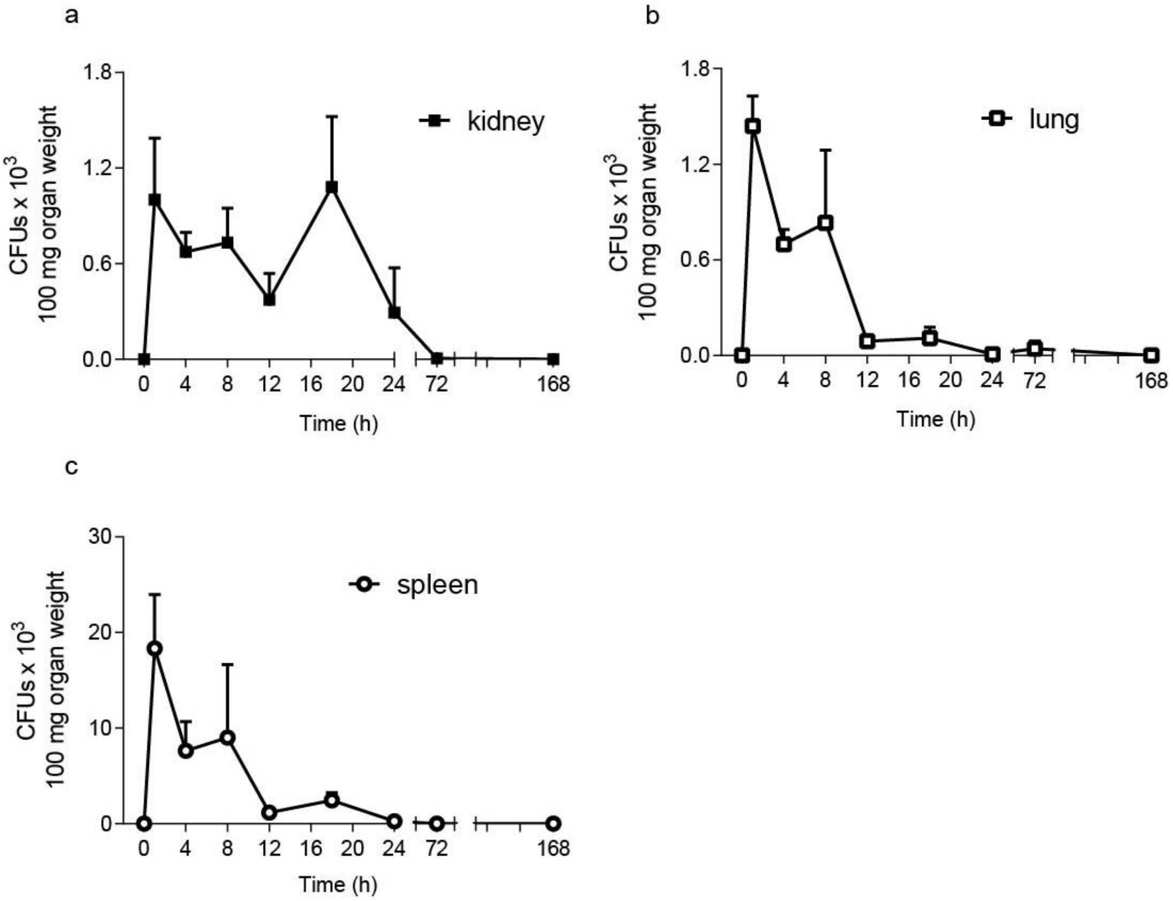

Supplementary Figure 1

Kinetic changes of the bacterial load in (**a**) **kidney,** (**b**) **lung, and** (**c**) **spleen of methicillin-resistant *Staphylococcus aureus* (MRSA)-infected mice monitored over a period of 7 days**. Data are representative of three experiments and are expressed as colony-forming units (CFUs). Data are shown as mean ± SD; n = 3 per group.

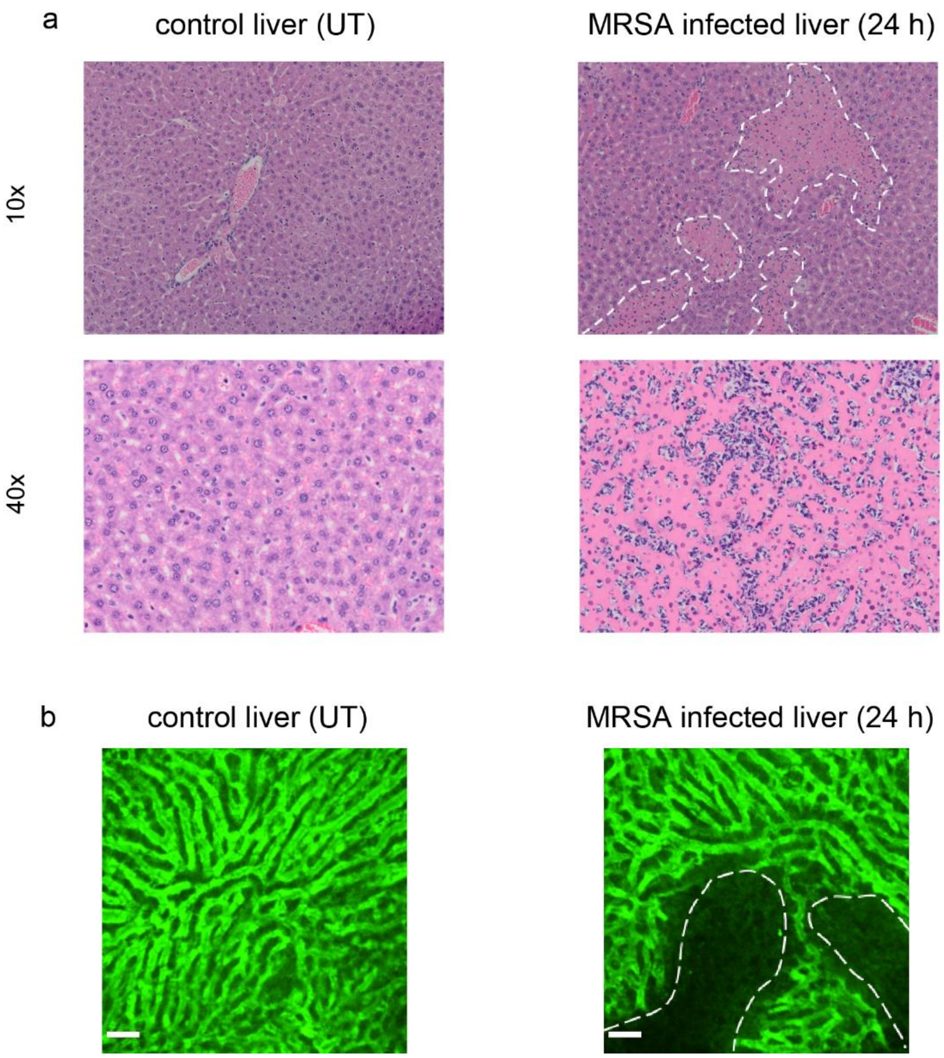

Supplementary Figure 2

Systemic infection with MRSA leads to development of non-perfused focal necrotic loci in the liver. (**a**) Representative fields of view from hematoxylin and eosin-stained liver tissue sections from control (untreated, UT) and MRSA-infected mice (24 h). Scale bars represent 100 µm (10x) and 25 µm (40x). Dashed line indicates borders of altered tissue forming focal necrotic loci. (**b**) Perfusion of liver sinusoids was tested by intravenous injection of FITC-labelled albumin into control (UT) and MRSA-infected mice (24 h) and recorded by IVM. The borders of necrotic areas are marked with a dashed line. The scale bar indicates 50 µm.

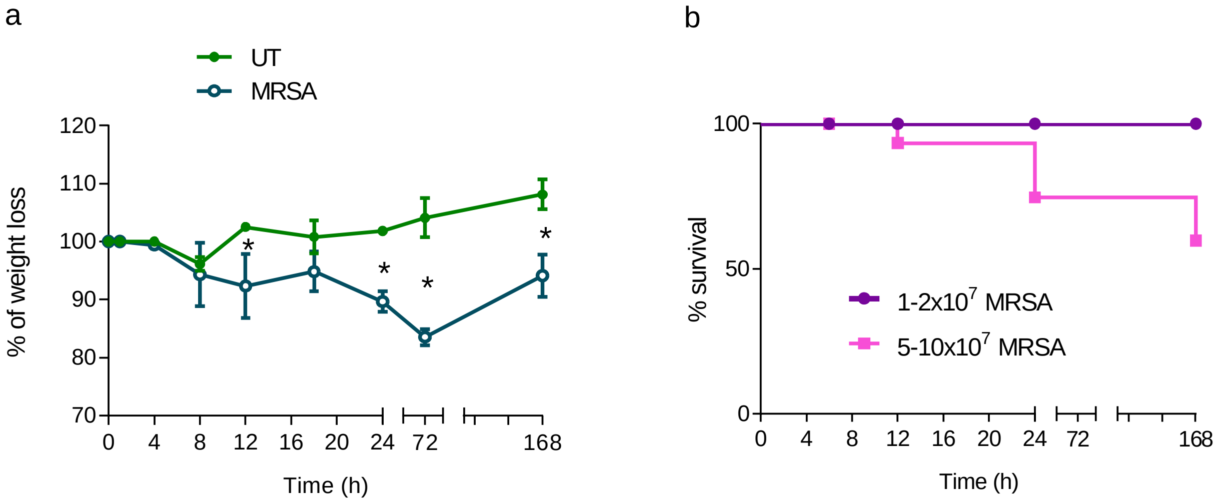

Supplementary Figure 3

Systemic infection with MRSA affects body weight and survival of mice. (**a**) Body weight of control (untreated, UT) and infected mice was monitored for 7 days, $^{*}0.01 < P \leq 0.05$ (t-test; comparison between UT and MRSA at each time point). (**b**) Survival of mice infected with different doses of MRSA, 1-2x10^7 *versus* 5-10x10^7 colony-forming units (CFUs). $P < 0.05$ (log-rank test). Data for weight are shown as mean ± SD; n = 3 (weight) and n = 5 (survival) per group.

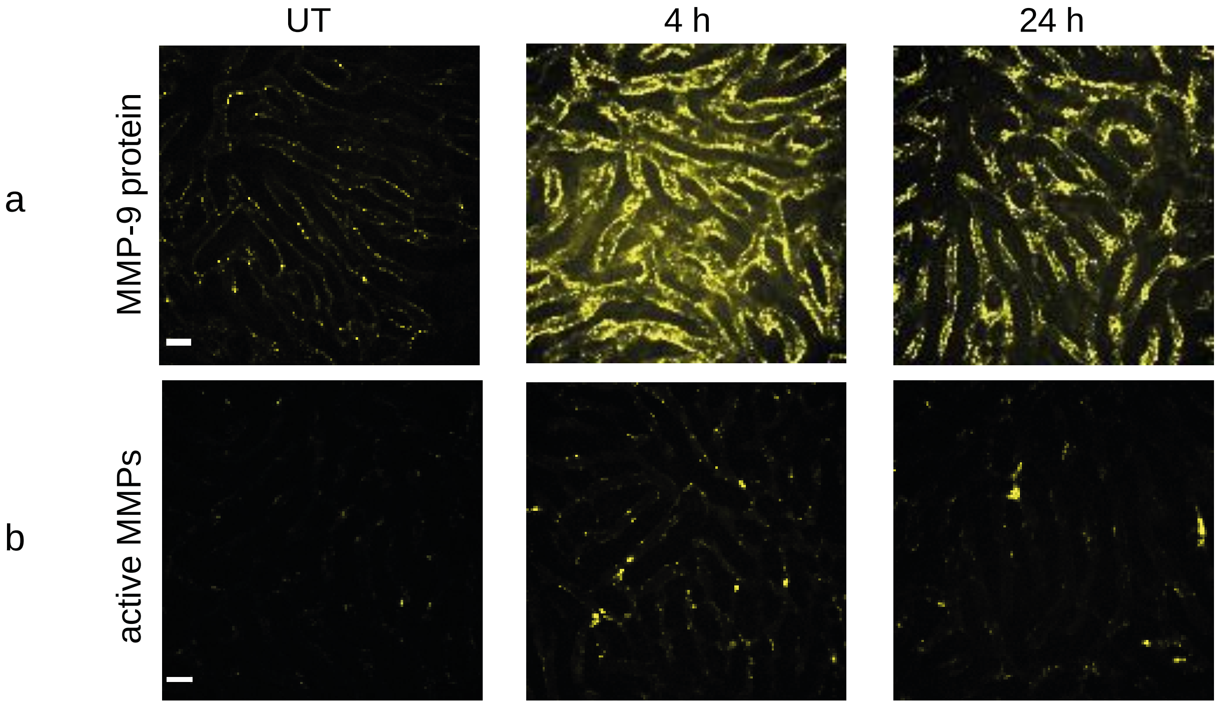

Supplementary Figure 4

Matrix metalloproteinase 9 (MMP-9) is attached to NETs but is proteolytically silent. (**a**) SD-IVM representative images of MMP-9 lining liver sinusoids. Images were obtained from untreated (UT) mice, and animals injected with MRSA, 4 and 24 h prior to imaging. MMP-9 was stained with AF647-anti-MMP-9 antibody (yellow; shown in false color to distinguish from the blue color code used for NE). The scale bar indicates 20 µm. Activity of MMP was measured by *in vivo* zymography in which otherwise silent substrate becomes fluorescent in the presence of active MMPs: (**b**) Representative images of liver sinusoids laid with processed substrate indicative of active MMPs (shown in false color). The scale bar indicates 20 µm. Data are representative of three experiments.

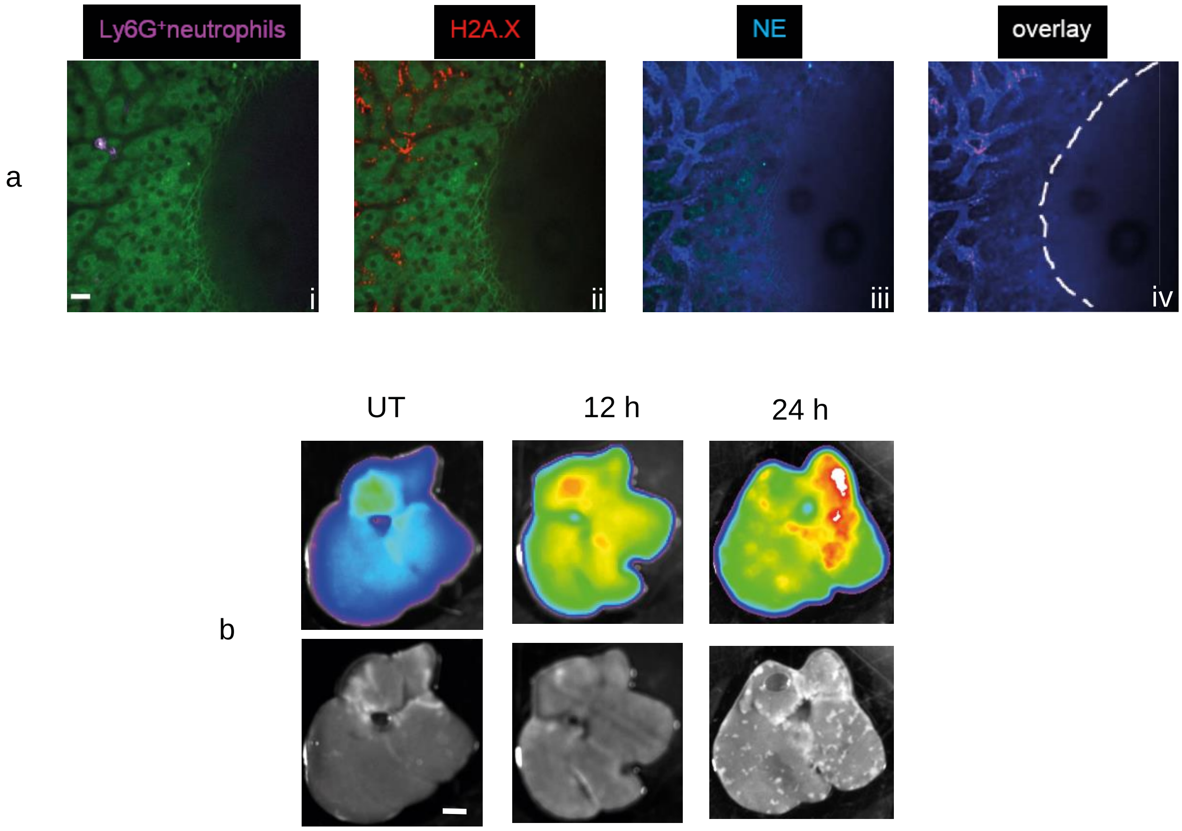

Supplementary Figure 5

Hotspots of active neutrophil elastase (NE) start to form before necrotic loci can be observed on the liver surface. (**a**) Representative images of NETs were acquired with SD-IVM 24 h post MRSA intravenous injection. (i) Neutrophils (magenta) were stained with A750-Ly-6G antibody, (ii) histones H2A.X were detected with AF555-anti-H2A.X antibody (red) and (iii) neutrophil elastase (NE) with AF647-anti-NE antibody (blue). Dashed line indicates position of the necrotic area (iv; overlay of ii-iii, the green channel was removed). The scale bar indicates 20 µm. (**b**) Representative images of livers collected from healthy mice (untreated, UT) or injected with MRSA, 12 and 24 h prior to imaging. The appearance of NE hotspots can be seen prior to development of necrotic loci (12 vs. 24 h). The scale bar indicates 5 mm.

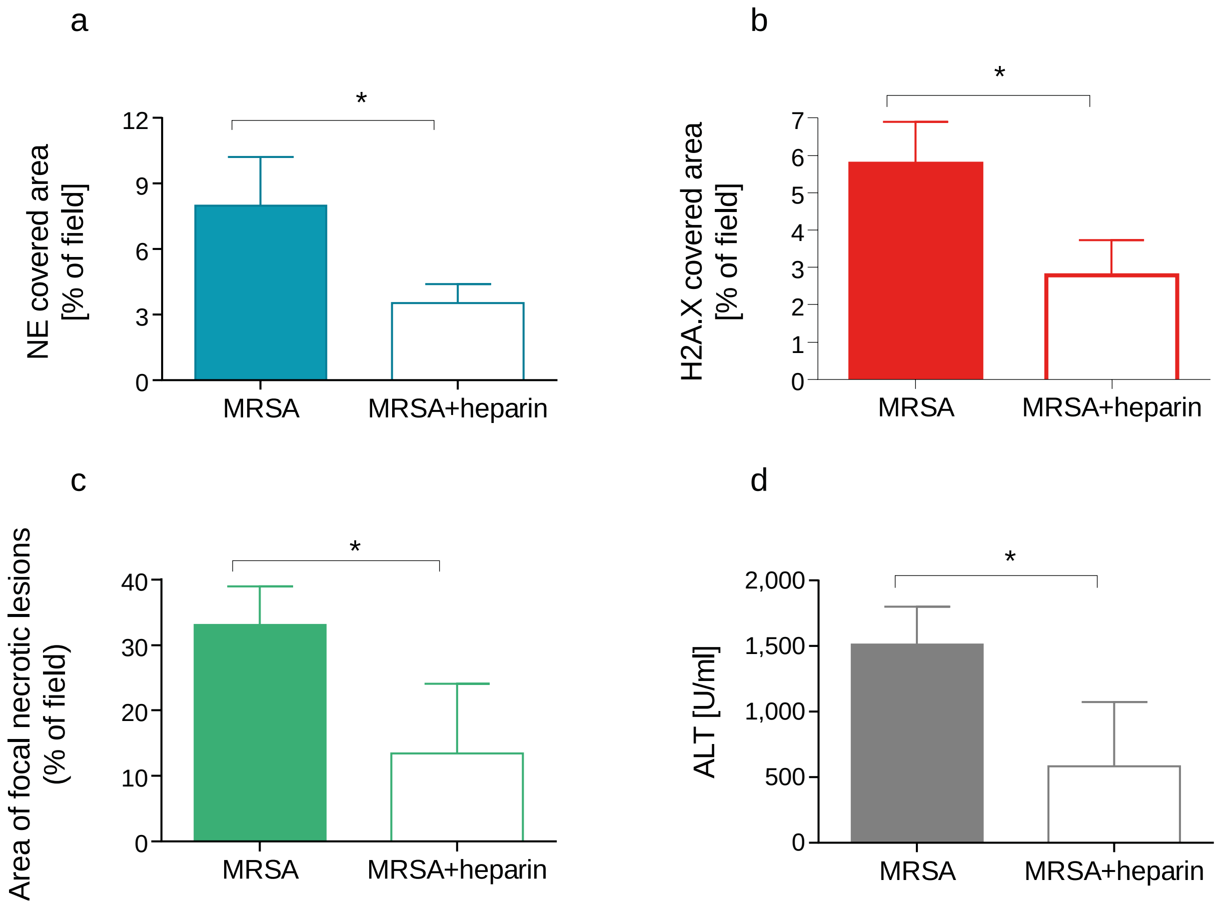

Supplementary Figure 6

Heparin acts in a similar manner as DNase. Non-fractionated heparin was applied by s.c. injection at the time of MRSA inoculation and again 12 h later, and compared to s.c. saline treated mice (MRSA+heparin *versus* MRSA). NET area covered by (**a**) NE and (**b**) H2A.X staining was quantified 4 h after MRSA injection. (**c**) The area of the liver covered with altered tissue was evaluated by ImageJ, and (**d**) serum ALT levels were measured 24 h post MRSA inoculation. $^{*}0.01 < P \leq 0.05$ (t-test). Data are shown as mean ± SD; n = 3 per group.

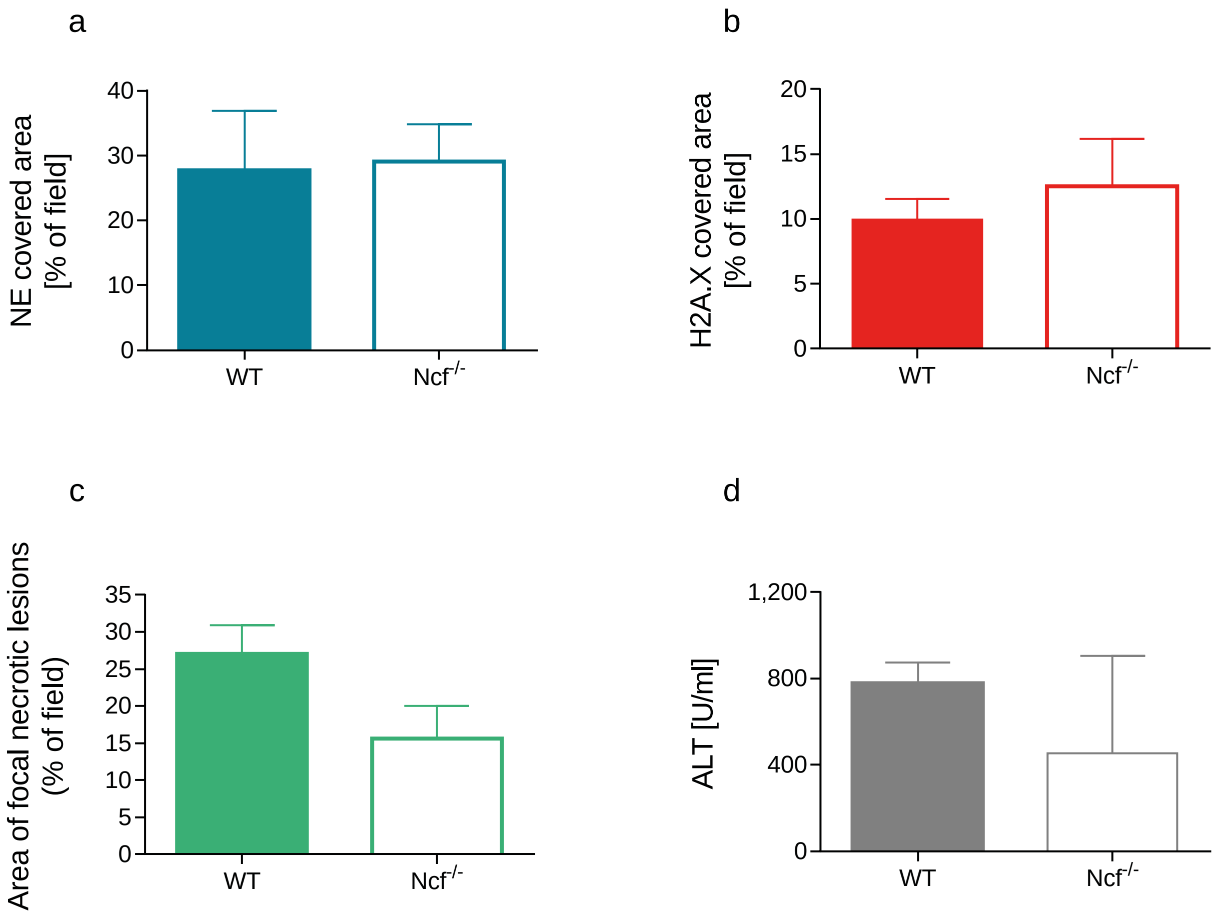

Supplementary Figure 7

NET formation is independent of NADPH oxidase-controlled reactive oxygen species. Quantitative analysis of NETs within the livers of MRSA infected wild-type (WT) and Ncf-deficient ($Ncf^{-/-}$) mice at 4 h of sepsis: (**a**) areas of NE and (**b**) of H2A.X staining. (**c**) Changes in liver morphology in $Ncf^{-/-}$ and WT mice evaluated by ImageJ as the area covered with necrotic tissue, and (**d**) serum ALT levels, both parameters were evaluated/measured in septic mice at 24 h post MRSA inoculation. Data are shown as mean ± SD; n = 2-4 per group.

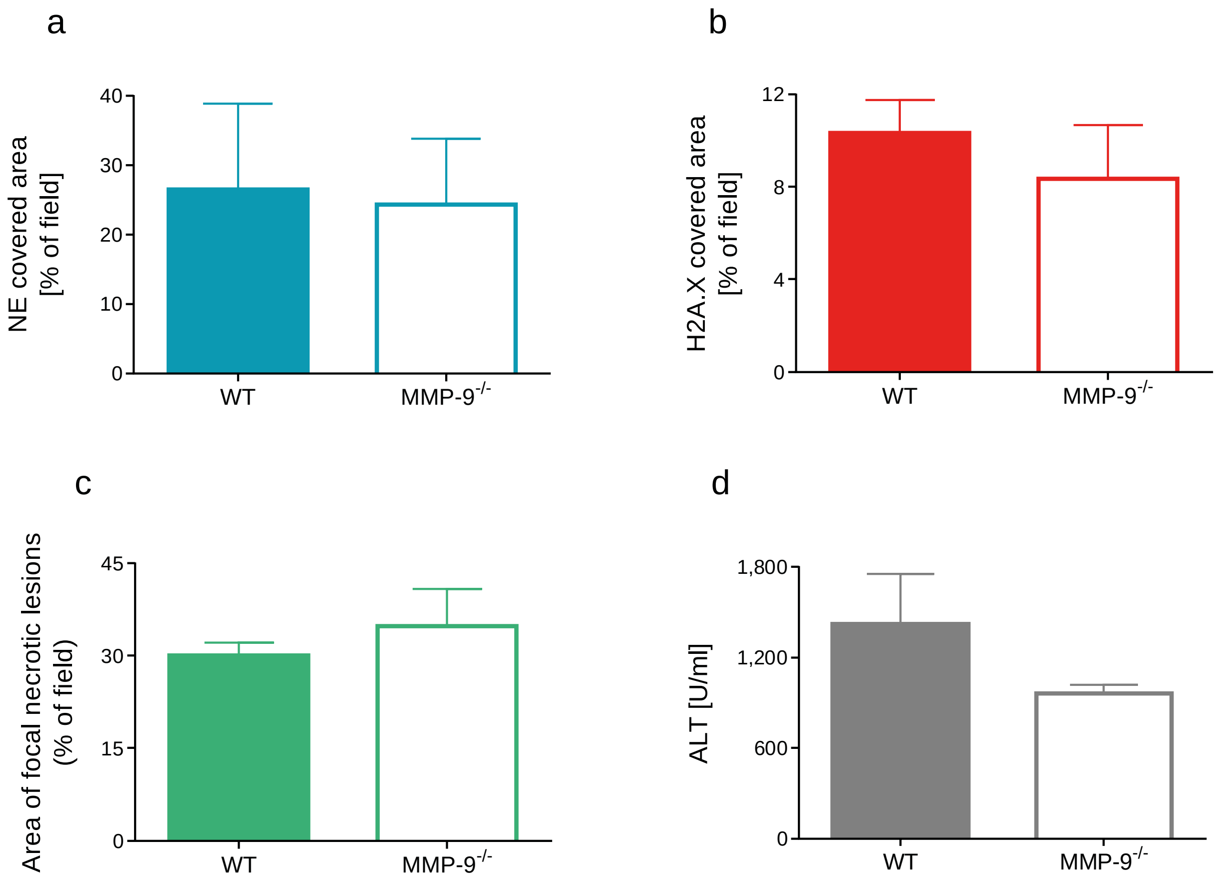

Supplementary Figure 8

MMP-9 is not indispensable for either NET formation or hepatic damage during MRSA sepsis. Quantitative analysis of NETs within the livers of MRSA infected wild-type (WT) and MMP-9 deficient (MMP-$9^{-/-}$) mice at 4 h of sepsis: (**a**) areas of NE and (**b**) of H2A.X staining. (**c**) Changes in liver morphology evaluated by ImageJ as the area covered with necrotic loci, and (**d**) serum ALT levels, both parameters were evaluated/measured in septic MMP-$9^{-/-}$ and WT mice at 24 h post MRSA inoculation. Data are representative of three experiments. Data are shown as mean ± SD; n = 3-4 per group. $^{*}0.01 < P \leq 0.05$ (t-test).

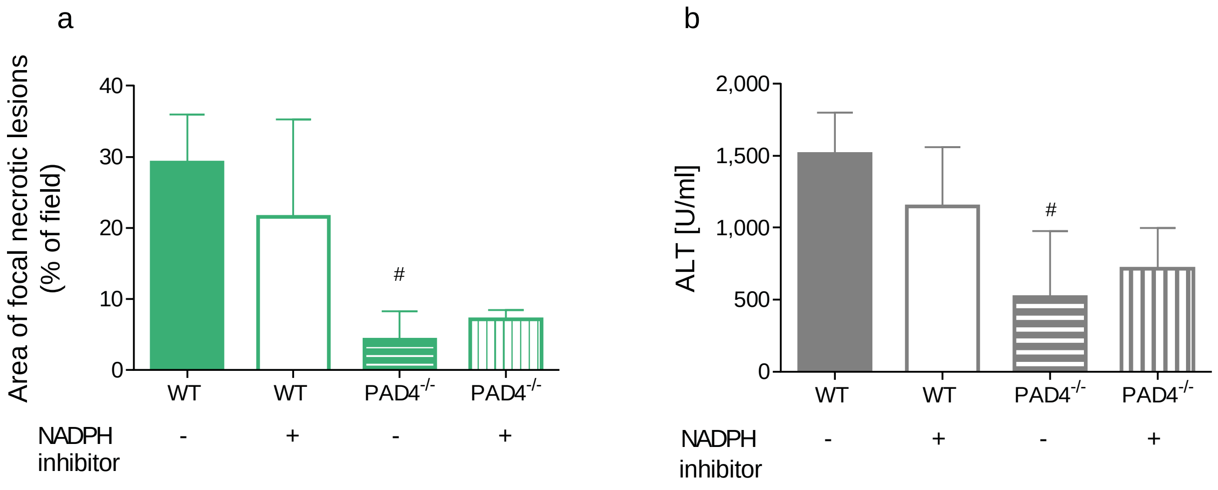

Supplementary Figure 9

MRSA-induced damage is not caused by NADPH-dependent reactive oxygen species. WT mice and PAD4-deficient mice ($PAD4^{-/-}$) were pretreated with NADPH oxidase inhibitor (apocynin) and the damage to their livers was assessed 24 h after MRSA injection: (**a**) changes in liver morphology, and (**b**) ALT levels. Data are shown as mean ± SD; n = 2-5 per group. $^{\#}0.01 < P \leq 0.05$ (t-test; a difference from inhibitor untreated WT mice).

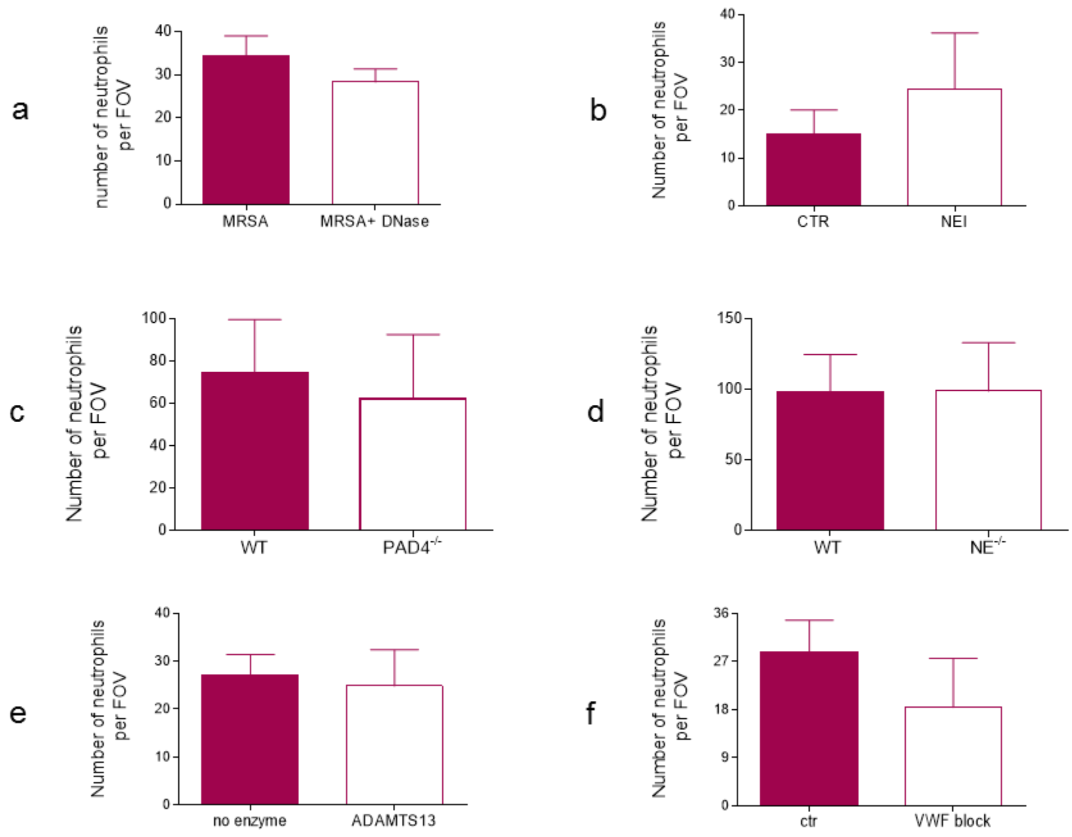

Supplementary Figure 10

Accumulation of neutrophils in liver sinusoids after intravenous inoculation of MRSA in different groups of transgenic mice and/or upon diverse treatments applied to C57Bl/6 mice. Numbers of infiltrating neutrophils were imaged with intravital microscopy (10x magnification) and quantified with ImageJ software, and are expressed as number per field of view (FOV). (**a**) Mice with ongoing MRSA sepsis were i.v. injected with DNase at 4 hrs and neutrophil numbers were counted at 24 hrs; (**b**) Some animals with ongoing MRSA sepsis were i.v. injected with neutrophil elastase inhibitor (NEI) at 4 hrs and neutrophil numbers were evaluated at 24 hrs; PAD4$^{-/-}$ mice (**c**) and neutrophil elastase knockout mice (NE$^{-/-}$) (**d**) were injected with MRSA and imaged 4 hours later for neutrophil counts evaluation. (**e**) Another group of animals that was i.v. injected with MRSA was treated with ADAMTS13 eight hours later, neutrophil counts were performer at 24 hrs. (**f**) Some mice were pretreated with an anti-von Willebrand factor (VWF) antibody before MRSA inoculation, and neutrophil counts were performer 24 hrs later.

Supplementary Figure 11

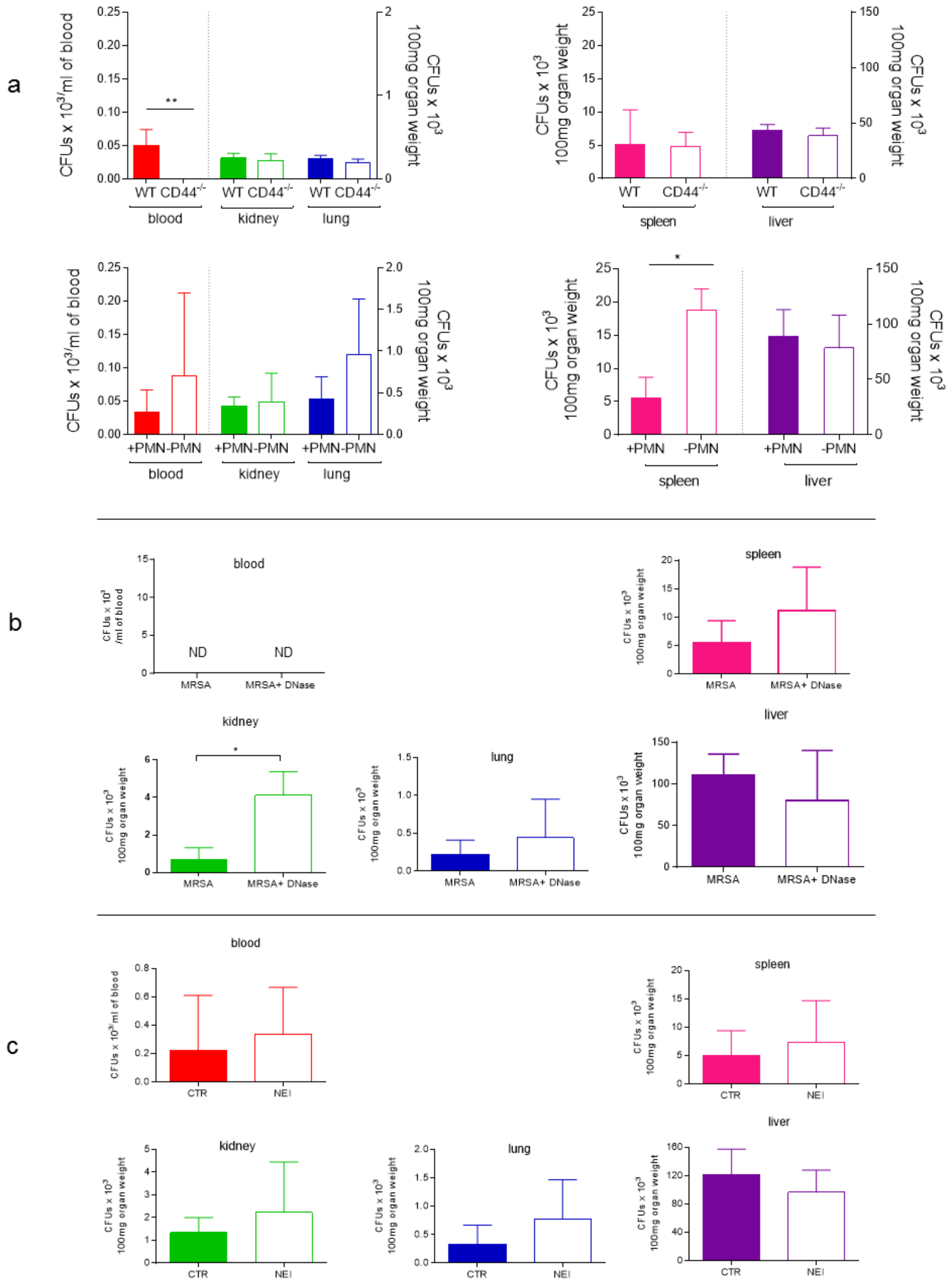

d

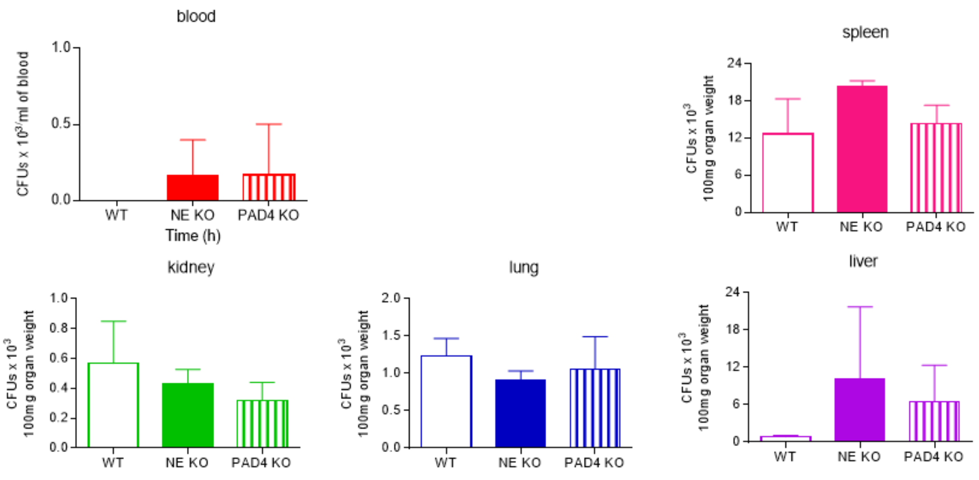

e

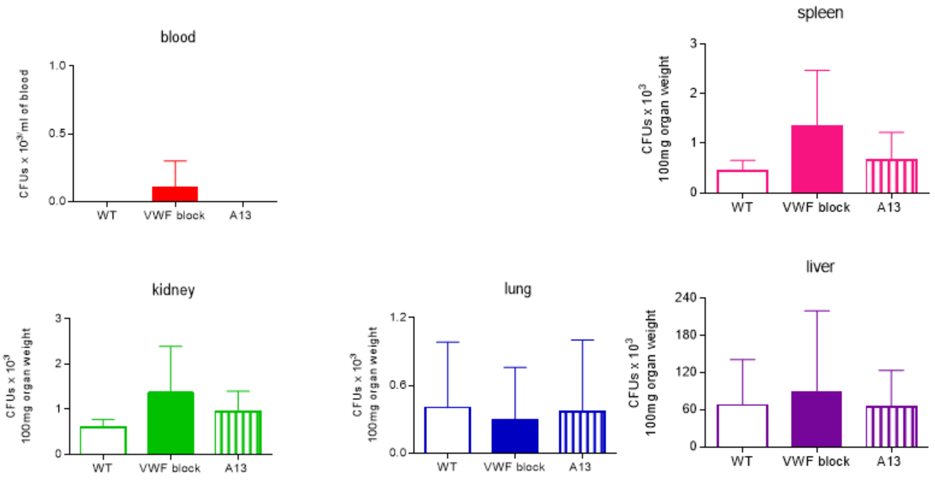

Supplementary Figure 11

Bacterial load in the blood and liver, spleen, kidney, and lung of MRSA-infected transgenic mice and/or upon diverse treatments applied to C57Bl/6 mice. All groups of mice were inoculated with MRSA and the data on bacterial load are expressed as colony forming units (CFUs). (a) some mice were depleted of neutrophils (-PMN) and their CFUs were compared to animals with normal neutrophil population (+PMN); in addition CFUs of CD44-/- mice were compared to CFUs of wild-type controls at 24 hrs of sepsis. (b) a group of mice with ongoing MRSA sepsis was i.v. injected with DNase at 4 hrs and CFUs were counted at 24 hrs; (c) another group of animals with ongoing MRSA sepsis was i.v. injected with neutrophil elastase inhibitor (NEI) at 4 hrs and CFUs were evaluated at 24 hrs. (d) Neutrophil elastase knockout mice (NE-/-) and PAD4-/- mice were injected with MRSA and 4 hours later CFUs were evaluated. (e) Another group of animals that was i.v. injected with MRSA was treated with ADAMTS13 8 hours later. Alternatively, Mice were pretreated with an anti-von Willebrand factor (VWF) antibody before MRSA inoculation. In both cases the CFUs were counted 24 hrs later.

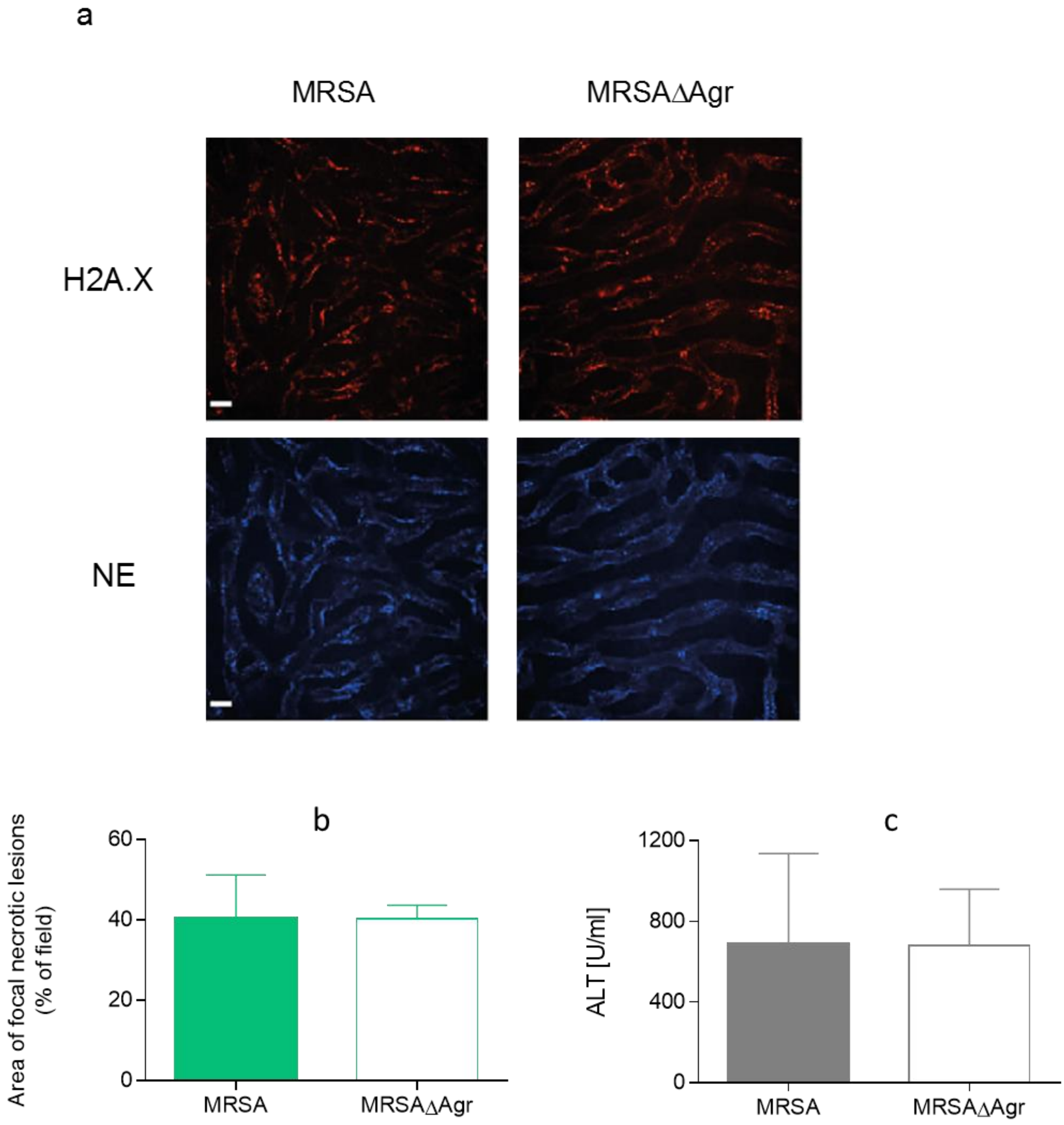

Supplementary Figure 12

MRSA toxins do not contribute to the liver damage. (**a**) Representative pictures of NET staining within the livers of wild-type MRSA infected mice, and mice infected with Agr knockout MRSA at 24 h of sepsis: histones (H2A.X) and neutrophil elastase (NE). (**b**) Changes in liver morphology evaluated by ImageJ as the area covered with necrotic loci, and (**c**) serum ALT levels; both parameters were evaluated/measured in mice infected with MRSA and MRSAΔAgr at 24 h post bacteria inoculation. Data are shown as mean ± SD. The scale bar indicates 20 μm.
